# Investigating the Associations between Drought, Poverty, High-Risk Sexual Behaviours, and HIV Incidence in Sub-Saharan Africa: A Cross-Sectional Study

**DOI:** 10.1007/s10461-024-04280-8

**Published:** 2024-02-20

**Authors:** Adam Trickey, Leigh F. Johnson, Rogerio Bonifacio, Agnes Kiragga, Guy Howard, Samuel Biraro, Thorsten Wagener, Andrea Low, Peter Vickerman

**Affiliations:** 1 Population Health Sciences, University of Bristol, Bristol, UK; 2 Centre for Infectious Disease Epidemiology and Research, School of Public Health and Family Medicine, University of Cape Town, Cape Town, South Africa; 3 Geospatial Analysis Unit, World Food Programme, Rome, Italy; 4 Research Department, Infectious Diseases Institute, College of Health Sciences, Makerere University, Kampala, Uganda; 5 Department of Civil Engineering and Cabot Institute of the Environment, University of Bristol, Bristol, UK; 6 ICAP at Columbia University, Nakasero, Kampala Uganda; 7 Institute of Environmental Science and Geography, University of Potsdam, Potsdam, Germany; 8 Department of Epidemiology, Mailman School of Public Health, Columbia University, New York, NY USA; 9 NIHR Health Protection Research Unit in Behavioural Science and Evaluation at University of Bristol, Bristol, UK

**Keywords:** Incidence, AIDS, Rainfall, Climate, Serosurvey, Structural factors

## Abstract

Climate change is increasing the likelihood of drought in sub-Saharan Africa, where HIV prevalence is high. Drought could increase HIV transmission through various mediating mechanisms; we investigated these associations. We used data on people aged 15–59 from Population-Based HIV Impact Assessment surveys from 2016 in Eswatini, Lesotho, Tanzania, Uganda, and Zambia. Survey data were geospatially linked to precipitation data for 2014–2016, with local droughts defined as cumulative rainfall between 2014 and 2016 being in < 15th percentile of all 2-year periods over 1981–2016. Using multivariable logistic regression, stratified by sex and rural/urban residence, we examined associations between (a) drought and poverty, (b) wealth quintiles and sexual behaviours (transactional, high-risk, and intergenerational sex), (c) sexual behaviours and recently acquiring HIV, and (d) drought and recent HIV. Among 102,081 people, 31.5% resided in areas affected by drought during 2014–2016. Experiencing drought was positively associated with poverty for women and men in rural, but not urban, areas. For each group, increasing wealth was negatively associated with transactional sex. For rural women, intergenerational sex was positively associated with wealth. Women reporting each sexual behaviour had higher odds of recent HIV, with strong associations seen for high-risk sex, and, for urban women, intergenerational sex, with weaker associations among men. Women in rural areas who had been exposed to drought had higher odds of having recently acquired HIV (2.10 [95%CI: 1.17–3.77]), but not women in urban areas, or men. Droughts could potentially increase HIV transmission through increasing poverty and then sexual risk behaviours, particularly among women in rural areas.

**Supplementary Information:**

The online version contains supplementary material available at 10.1007/s10461-024-04280-8.

## Background

Climate change is hypothesised to be impacting the HIV epidemic in settings where Human immunodeficiency virus (HIV) prevalence is high [1]. Climate change is increasing severe weather events, including heatwaves, flooding and cyclones, as well as meteorological droughts [2, 3], defined as an exceptional lack of water compared to normal circumstances [4]. In turn, these weather events are impacting on health through various mechanisms. These include heat-related illnesses [5], and altered patterns of vector-borne and water-borne diseases linked to drought [6–9]. Sub-Saharan Africa (SSA) contains the majority of the world’s HIV burden [10]. SSA will also be one of the regions most affected by climate change, with increasing risks of drought caused by changes in precipitation and limited water storage, as well as limited capacity and resources to support adaptation. Drought is an ongoing and worsening trend [3], with the fraction of SSA experiencing severe drought increasing from < 5% to ~ 15% since 1901 [11].

The hypothesised impact of climate change on HIV occurs through various intermediary mechanisms. Extreme weather events, such as droughts, cause decreased food yields [12], which impact negatively on human health, increasing poverty and food insecurity, and exacerbating the structural problems underlying HIV transmission, particularly among women [10, 13–18]. This can be through changes in sexual behaviours driven by poverty, such as increased transactional sex [19], which is also linked to intergenerational sex - particularly where young women partner with older men who have more resources and higher HIV prevalence [13, 14, 20, 21]. Women in these circumstances may have less say in the use of contraception, increasing condomless sex with casual partners [22]. Additionally, drought has been hypothesised to affect HIV acquisition through an increase in gender-based violence and worsening mental health, which can impact sexual behaviours and access to HIV prevention services [18, 23]. There are other links between climate change and HIV. Migration due to severe weather events could also bring new population groups together, increasing transmission of viruses [24], and potentially disrupting the provision of antiretroviral therapy (ART) due to a lack of access or competing priorities. For some regimens, ART absorption can also decrease in the absence of food [25, 26].

The Joint United Nations Programme on HIV/AIDS (UNAIDS) warned in 2008 that global warming could have a detrimental effect on HIV transmission, yet at the time there had been little research on these effects [1]. Since that warning, epidemiological analyses have found that rural areas of sub-Saharan Africa with increased droughts or flooding events have elevated HIV prevalence [16, 27]. Meanwhile, modelling has estimated that climate change may result in 11.6–16.0 million additional cases of HIV by 2050 without action to reduce emissions [28]. Other analyses have shown associations between droughts and reduced HIV testing in the prior year as well as heightened HIV prevalence and sexual behaviours [29–31]. Otherwise, research on the association between poverty and HIV in the SSA context has usually not considered drought and climate change [23, 32]. The relationships between poverty and HIV in SSA are nuanced and debated; at a global level HIV is thought to disproportionately affect poorer individuals [33], whilst in SSA the relationship between poverty and HIV differs between urban and rural areas [34]. More research is required on the mechanisms by which poverty affects HIV, and, additionally, on the associations connecting drought with HIV via poverty.

We aimed to address this research gap by investigating a potential pathway by which drought may affect HIV transmission through examining associations between drought and poverty, poverty and sexual behaviours, and sexual behaviours and HIV incidence, as well as associations between drought and HIV incidence.

## Methods

**Fig. 1:**
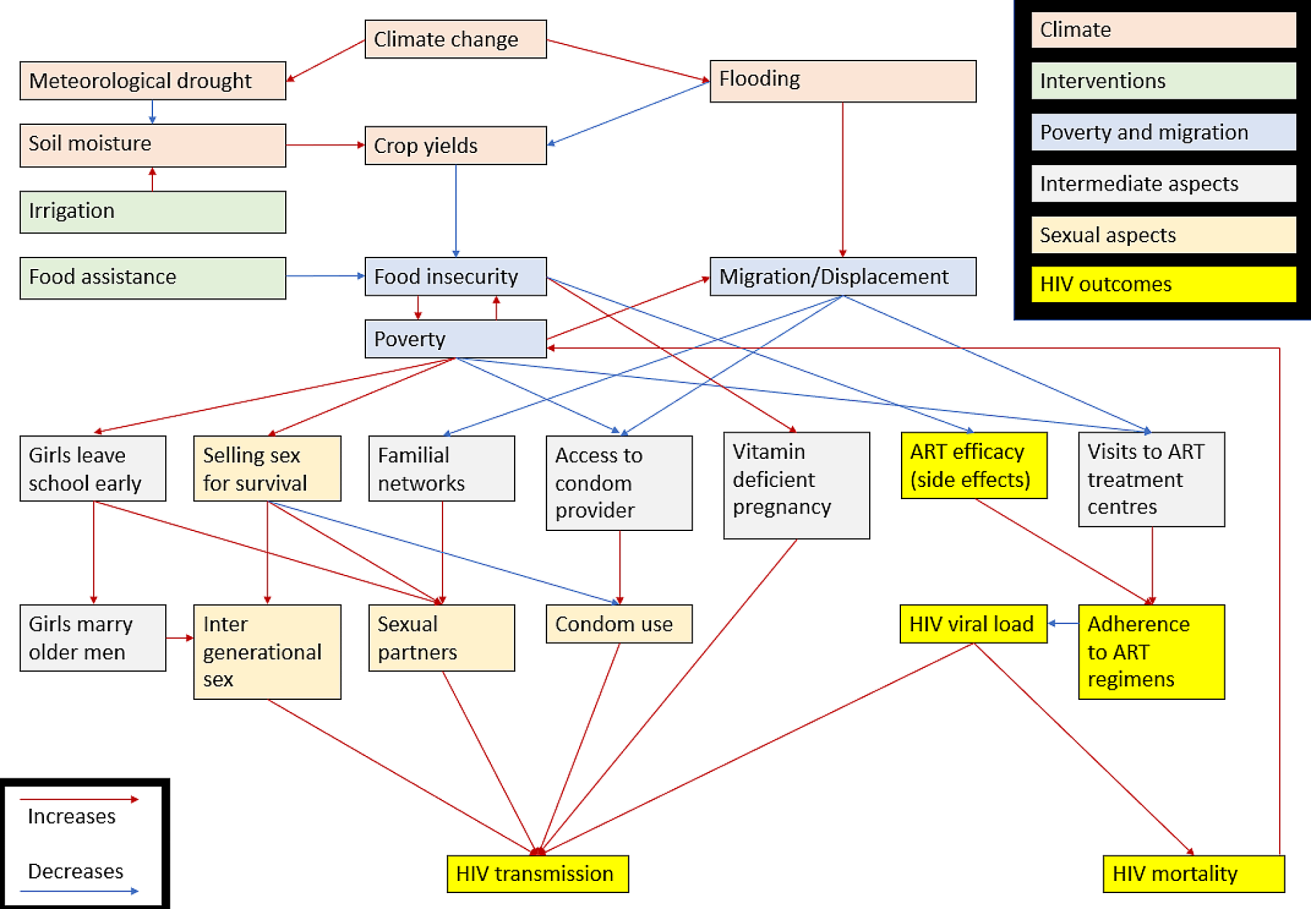
Theoretical framework linking climate change and drought with HIV transmission

A diagram of the theorised connections between climate change and HIV is shown in Fig. 1.

### Data and Procedures

Data were taken from five nationally representative Population-Based HIV Impact Assessment (PHIA) cross-sectional surveys where data collection started in 2016 (Eswatini, Lesotho, Tanzania, Uganda, and Zambia). People were surveyed using a two-stage sampling design, stratified by urban and rural areas, as described previously [35, 36]. Consent was indicated by signing or making a mark on the consent form on a tablet computer and on a printed copy, which was retained by the participant. Further details on sampling, eligibility, consent procedures, staff and training, data collection and cleaning, and response rates are provided in the individual country final reports. Links to these reports are given on page 3 and in supplementary Tables 1–5 of the appendix and include the questionnaires used. In each survey people aged 0–59 years (or greater) were sampled, however, in this analysis we only include those aged 15–59 years old, so that adults of the same age were analysed across all surveys. We also excluded individuals with missing data on wealth quintile or education. Missing data were not imputed as they were rare (< 1% for most variables) and this would have added considerable complexity to the analyses and limited the outputs that could be produced.

The household questionnaire captured data from the household head on assets and receipt of social support in the past 3 months, whilst the adult questionnaire was administered to all consenting eligible participants aged ≥ 15 years during face-to-face interviews. The adult questionnaire included questions on demographics, lifetime and recent (over the past 12 months) sexual behaviours, and on characteristics of their three most recent sexual partners. Further details on survey questions are provided in the online supplemental appendix.

Participants were tested for HIV by survey staff using the national testing algorithm. Among adults that tested positive for HIV, real-time polymerase chain reaction was used to measure HIV ribonucleic acid (RNA) in plasma and dried blood spots (DBS), and qualitative screening was done on DBS specimens to detect the most common antiretroviral drugs with long half-lives. To classify recent infections among adults, staff used HIV-1 limited antigen avidity immunoassays. Samples were considered a recent infection if there was a normalised optical density < 1.5 and the individual did not have a suppressed viral load (HIV-1 RNA < 1000 copies/mL) or detectable antiretroviral drugs.

Data on precipitation estimates from the Climate Hazards Group InfraRed Precipitation with Station Data (CHIRPS) at 0.05° resolution were used create a variable comparing current and historical rainfall that was used to define drought [37]. This was a dataset prepared by the Vulnerability Analysis and Mapping Geospatial Analysis Team at the Analysis and Trends Service of the World Food Programme. The dataset summed the 2-year total rainfall from June 2014 to May 2016 for each gridded location, which was then ranked compared to all 2-year rainfall amounts in the same gridded location over the period 1981–2016 to give an empirical percentile. Further data on the climatic context preceding the surveys are given in the appendix. This 2-year period was chosen to align with Southern Africa having experienced a severe drought from late-2014 to mid-2016, including two separate rainfall seasons, October 2014-May 2015 and October 2015-May 2016. The previous and following seasons registered abundant rainfall and good levels of crop production, hence this 2014–2016 can be seen as an episodic but very intense drought period (supplementary Fig. 1, supplementary Table 6).

Each participant’s household location was included within an enumeration area based on the most recent census from each country. These enumeration areas are geographic areas that differ in size depending on population density. They are the smallest statistical sample unit in a national census and are usually defined by the number of households they contain, which is usually the number of households that a single staff member can visit and interview during the census period. As it is quicker to move between households when they are closer together, urban enumeration areas usually have more households than rural enumeration areas. Enumeration areas are normally smaller than the grids in each dataset, although very rural areas with small populations could be quite large.

We combined the PHIA survey data with the gridded data on drought using latitude and longitude data from the centroid of each primary sampling (enumeration area) unit in the surveys, to define whether that area had experienced drought.

### Variables

Drought was defined if the rainfall during the 2-year period (June 2014 to May 2016) was less than the 15th percentile compared with historical 2-year periods from 1981 to 2016 for the same location. This binary variable approximates the level below which rainfall deficits are particularly harmful to gross domestic product and maize yields [16]. We also performed sensitivity analyses, defining drought using the 10th percentiles.

All analyses were stratified by sex and rural/urban location to account for a higher prevalence of subsistence farming in rural areas [16] and differences in sexual behaviours between men and women. Variables used for adjustment were survey country, age, having received a secondary-level education or higher, whether they had ever been married, whether they had reported receiving a pay check in the last 2 months, whether they had experienced severe food insecurity (defined as having no food more than 3 time in the last 4 weeks [38]), and whether they had received economic support in the previous 3 months (no support, food support, or other economic support). This support could include cash transfers, assistance for school fees, education, or shelter, social pensions, or other.

Relative poverty, henceforth referred to as poverty, was defined as being in the lowest two wealth quintiles for each country’s survey. These wealth quintiles (described in detail on supplementary materials page 17) were also included as a continuous variable in the analyses with sexual behaviours as the outcome. Where age and wealth quintile were included as continuous variables, sensitivity analyses were also performed in which these were included as categorical variables.

The recent sexual behaviours considered were transactional sex, high-risk sex, and intergenerational sex. Each sexual behaviour could overlap with the others but we do not explicitly include transactional sex or intergenerational sex in high-risk sex. Transactional sex was defined as having bought/sold sex (unavailable for Zambia) or entered into a relationship to either supply or receive food, money, or support. Transactional sex was defined the same for both men and women in our analysis, but in practice most men engaging in transactional sex are supplying money/food/support, and most women engaging in transactional sex are receiving this [39]. High-risk sex was defined as condomless sex with a partner who was HIV + or had unknown HIV status, who they did not live with and were not married to. Intergenerational sex was defined for women as sex with a partner who was older by ≥ 10 years, and for men as sex with a partner that who was younger by ≥ 10 years. Intergenerational sex was defined differently for men and women because it mostly occurs between young women and older men [21]. Due to the imbalanced power dynamics, the women in these age-disparate relationships are less able to negotiate safe-sex practices [40], leading to high HIV incidence [41]. Additionally, there is literature from the Southern African region that shows a strong link between age-disparate relationships and partner concurrency [42, 43].

Regional viraemia was defined as the percentage of adults in the enumeration area with unsuppressed HIV-1 viral loads.

### Statistical Analyses

The analyses investigating the associations between drought and poverty were adjusted for demographic variables and variables regarding economic support, age, secondary education or higher, having ever been married, having received a paycheck in the last 12 months, receipt of food support or other economic support, and survey country. Univariable and multivariable odds ratios (ORs) were calculated using logistic regression, with household-level survey weights. All survey weighting was done using Taylor-series weights, accounting for clustering by sampling units [44].

For the analyses examining the associations between wealth quintiles and sexual behaviours (transactional sex, high-risk sex, and intergenerational sex), logistic regression was used with the individual-level survey weights. To assess the effect of variables that could impact household wealth, analyses were unadjusted, adjusted for age and survey country, and then fully adjusted for demographic variables, age, survey country, receipt of food or other economic support, secondary education or higher, and having received a paycheck in the previous 12 months, ever having been married.

In analyses of the associations between the sexual behaviours and recent HIV, logistic regression was used with the survey weighting for blood testing. The regression models contained each sexual behaviour (transactional sex, high-risk sex, and intergenerational sex) and were also adjusted for age, and an interaction term between survey country and regional HIV viraemia levels.

To investigate the associations between drought and recent HIV, we used logistic regression with the survey weighting for blood testing. We first used unadjusted models and then adjusted for potential mediators: age and an interaction term between survey country and regional HIV viraemia levels. We then further adjusted for demographic and sexual behaviour variables that could mediate the relationship between drought and recent HIV, age, an interaction term between survey country and regional viraemia, secondary education or higher, having received a paycheck in the previous 12 months, ever having been married, receipt of food or other economic support, severe food insecurity, wealth quintile, transactional sex, high-risk sex, and intergenerational sex. Sensitivity analyses were performed without the survey weighting.

People with missing data on HIV testing were excluded from the analyses with recent HIV as the outcome. People with missing data on a binary variable, e.g. having ever been married or reporting a sexual behaviour were assumed unexposed.

### Ethical Approval

All PHIA survey protocols, consent forms, screening forms, refusal forms, referral forms, recruitment materials, and questionnaires were reviewed and approved by in-country ethics and regulatory bodies and the institutional review boards of Columbia University Medical Center, Westat, and the United States Centers for Disease Control.

## Results

Across the five country surveys, response rates varied from 84.5% in Eswatini (*N* = 11673), 89.4% in Zambia (*N* = 21280), 93.0% in Lesotho (*N* = 12887), 94.8% in Tanzania (*N* = 33004), to 96.7% in Uganda (*N* = 29383). We excluded persons aged ≥ 60 years; 1437 in Eswatini, 3361 in Tanzania, and 997 in Uganda. We also excluded persons with missing data on wealth quintile or education level; 15 in Eswatini, 42 in Lesotho, 22 in Tanzania, 159 in Uganda, and 113 in Zambia. In total, 102,081 respondents aged 15–59 were included; 12,845 (12.6%) from Lesotho, 29,621 (29.0%) from Tanzania, 28,227 (27.7%) from Uganda, 10,221 (10.0%) from Eswatini, and 21,167 (20.7%) from Zambia. Further information on survey response rates, exclusions, and missing data are given on supplementary materials page 13. The unweighted percentage of people from each country who had experienced a drought varied from 357 (1.3%) in Uganda and 1,187 (4.0%) in Tanzania, to 12,455 (58.8%) in Zambia, 6,740 (65.4%) in Eswatini, and 12,061 (93.9%) in Lesotho; 32,800 (32.1%) in total. Of the 102,081 respondents included, 58,341 (57.2%) were female and 34,388 (33.7%) lived in urban areas.

### Drought and poverty

Table 1 shows the weighted demographic characteristics of women and men stratified by wealth quintiles. Wealth quintiles were positively correlated with having received a secondary education. The percentage of people living in urban areas increased with each wealth quintile, whilst a decreasing trend was seen for ever having been married, having received food support, or other economic support, and experiencing severe food insecurity. The percentage of participants who were female or had experienced drought was similar across the wealth quintiles.

**Table 1:** Weighted* demographic characteristics by wealth quintile, stratified by gender

Variable	Wealth quintile 1	Wealth quintile 2	Wealth quintile 3	Wealth quintile 4	Wealth quintile 5	Total
N women	10,261 (19%)	10,538 (19%)	11,259 (20%)	11,259 (20%)	12,202 (22%)	55,464 (100%)
Mean age (95% confidence interval)	30.6 (30.3–30.9)	30.9 (30.6–31.2)	30.8 (30.5–31.1)	29.9 (29.6–30.1)	28.7 (28.5–29.0)	30.1 (30.0-30.3)
Urban	471 (5%)	693 (7%)	2501 (22%)	6300 (56%)	10,608 (87%)	20,544 (37%)
Secondary education (or higher)	889 (9%)	1682 (16%)	2855 (25%)	4536 (40%)	7363 (60%)	17,305 (31%)
Received paycheck last 12 months	3040 (30%)	3342 (32%)	3728 (33%)	4230 (38%)	5176 (42%)	19,496 (35%)
Ever married	8470 (83%)	8340 (79%)	8492 (75%)	7958 (71%)	7469 (61%)	40,688 (73%)
Tested HIV + in survey	660 (6%)	751 (7%)	1107 (10%)	1295 (12%)	1088 (9%)	4897 (9%)
Food support	207 (2%)	157 (1%)	116 (1%)	111 (1%)	70 (1%)	660 (1%)
Other economic support	813 (8%)	728 (7%)	637 (6%)	498 (4%)	400 (3%)	3073 (6%)
Severe food insecurity	1802 (18%)	1520 (14%)	1215 (11%)	858 (8%)	526 (4%)	5912 (11%)
Drought	1305 (13%)	1408 (13%)	1451 (13%)	1297 (12%)	1390 (11%)	6844 (12%)
Transactional sex	1308 (13%)	1164 (11%)	1297 (12%)	1208 (11%)	1026 (8%)	6001 (11%)
High-risk sex	358 (3%)	429 (4%)	466 (4%)	446 (4%)	458 (4%)	2158 (4%)
Intergenerational sex	1730 (17%)	1729 (16%)	1910 (17%)	1602 (14%)	1624 (13%)	8586 (15%)
N men	10,538 (19%)	11,315 (20%)	11,315 (20%)	10,926 (20%)	11,370 (21%)	55,464 (100%)
Mean age (95% confidence interval)	29.9 (29.5–30.2)	30.4 (30.0-30.7)	30.4 (30.0-30.8)	29.6 (29.3–29.9)	29.5 (29.2–29.8)	30.0 (29.8–30.1)
Urban	424 (4%)	708 (6%)	2280 (20%)	5872 (54%)	9833 (86%)	19,113 (34%)
Secondary education (or higher)	1719 (16%)	2454 (22%)	3557 (31%)	5399 (49%)	7835 (69%)	20,960 (38%)
Received paycheck last 12 months	4997 (47%)	5834 (52%)	6295 (56%)	6865 (63%)	7587 (67%)	31,576 (57%)
Ever married	6889 (65%)	7055 (62%)	6783 (60%)	6102 (56%)	5901 (52%)	32,729 (59%)
Tested HIV + in survey	453 (4%)	540 (5%)	673 (6%)	696 (6%)	534 (5%)	2895 (5%)
Food support	192 (2%)	140 (1%)	140 (1%)	106 (1%)	69 (1%)	649 (1%)
Other economic support	720 (7%)	651 (6%)	494 (4%)	397 (4%)	351 (3%)	2612 (5%)
Severe food insecurity	1679 (16%)	1427 (13%)	1108 (10%)	740 (7%)	421 (4%)	5374 (10%)
Drought	1233 (12%)	1504 (13%)	1495 (13%)	1283 (12%)	1393 (12%)	6905 (12%)
Transactional sex	408 (4%)	428 (4%)	413 (4%)	358 (3%)	299 (3%)	1908 (3%)
High-risk sex	625 (6%)	815 (7%)	824 (7%)	857 (8%)	661 (6%)	3783 (7%)
Intergenerational sex	1778 (17%)	1862 (16%)	2026 (18%)	1652 (15%)	1608 (14%)	8924 (16%)

*Household survey weighting

Table 2 shows the unadjusted and adjusted odds ratios of experiencing poverty. For women in rural areas, those who had experienced a recent drought had higher odds of being in poverty than those who had not (adjusted odds ratio [aOR] 1.28 [95% confidence interval: 1.02–1.61]). There was not strong evidence of a positive association for women in urban areas (1.24 [0.68–2.26]). Similarly, men in rural areas who had experienced a drought had higher odds of poverty (1.31 [1.04–1.66]), but not in urban areas (1.03 [0.46–2.27]).

**Table 2:** Weighted odds ratios of poverty*

		Outcome: Poverty			
		Rural		Urban	
**Women**		**Univariable**	**Multivariable**	**Univariable**	**Multivariable**
	Drought	1.34 (1.14–1.58)	1.28 (1.02–1.61)	0.56 (0.38–0.83)	1.24 (0.68–2.26)
	Age (per 10-year increase)	1.02 (0.99–1.04)	0.86 (0.83–0.89)	1.18 (1.11–1.26)	1.00 (0.94–1.06)
	Secondary education or higher	0.27 (0.24–0.30)	0.25 (0.23–0.28)	0.17 (0.13–0.23)	0.14 (0.11–0.19)
	Ever married	1.57 (1.44–1.70)	1.37 (1.22–1.53)	1.79 (1.47–2.16)	1.21 (0.99–1.49)
	Received paycheck in last 12 months	0.76 (0.71–0.82)	0.82 (0.76–0.88)	0.91 (0.76–1.10)	0.74 (0.61–0.90)
	Food support (vs. no support)	1.78 (1.21–2.62)	1.71 (1.42–2.05)	1.24 (0.60–2.53)	0.83 (0.39–1.78)
	Other economic support (vs. no support)	1.55 (1.28–1.87)	1.97 (1.32–2.92)	3.35 (2.40–4.68)	2.95 (2.03–4.29)
	Eswatini	1	1	1	1
	Lesotho	1.41 (1.16–1.72)	1.16 (0.94–1.43)	0.20 (0.13–0.31)	0.12 (0.08–0.20)
	Tanzania	1.49 (1.20–1.85)	1.00 (0.77–1.29)	0.20 (0.12–0.32)	0.08 (0.05–0.15)
	Uganda	1.05 (0.89–1.24)	0.80 (0.62–1.04)	0.56 (0.37–0.85)	0.33 (0.19–0.57)
	Zambia	1.65 (1.40–1.95)	1.17 (0.95–1.43)	0.07 (0.04–0.12)	0.04 (0.02–0.07)
**Men**		**Univariable**	**Multivariable**	**Univariable**	**Multivariable**
	Drought	1.24 (1.05–1.47)	1.31 (1.04–1.66)	0.46 (0.30–0.69)	1.03 (0.46–2.27)
	Age (per 10-year increase)	0.99 (0.97–1.02)	0.84 (0.81–0.87)	1.10 (1.02–1.18)	1.08 (0.96–1.21)
	Secondary education or higher	0.35 (0.32–0.38)	0.34 (0.31–0.37)	0.26 (0.19–0.35)	0.21 (0.16–0.29)
	Received paycheck in last 12 months	0.71 (0.66–0.76)	0.70 (0.65–0.76)	0.76 (0.58–1.01)	0.61 (0.45–0.83)
	Ever married	1.25 (1.18–1.33)	1.56 (1.43–1.71)	1.22 (0.97–1.52)	0.87 (0.60–1.25)
	Food support (vs. no support)	1.44 (0.94–2.18)	1.63 (1.06–2.52)	0.69 (0.28–1.72)	0.55 (0.22–1.38)
	Other economic support (vs. no support)	1.57 (1.27–1.93)	1.77 (1.44–2.19)	2.93 (2.05–4.17)	2.71 (1.81–4.06)
	Eswatini	1	1	1	1
	Lesotho	1.54 (1.27–1.88)	1.14 (0.92–1.41)	0.18 (0.11–0.28)	0.13 (0.08–0.23)
	Tanzania	1.58 (1.27–1.97)	1.32 (1.03–1.70)	0.20 (0.12–0.33)	0.12 (0.07–0.23)
	Uganda	1.17 (0.98–1.39)	1.15 (0.89–1.48)	0.61 (0.41–0.92)	0.52 (0.29–0.93)
	Zambia	1.53 (1.28–1.81)	1.38 (1.13–1.68)	0.05 (0.03–0.08)	0.05 (0.03–0.08)

*Poverty defined as being in the lowest two wealth quintiles

In sensitivity analyses using a more stringent definition of drought (< 10th percentile of rainfall instead of < 15th percentile), the association between drought and poverty for women and men in rural areas remained (aORs 1.42 [1.17–1.73] and 1.43 [1.15–1.76], respectively), whilst it was attenuated for women and men in urban areas (1.50 [0.69–2.31] and 1.43 [0.86–1.36], respectively). In sensitivity analyses treating age as a categorical variable (supplementary Table 7), results were similar.

### Poverty and Sexual Behaviours

Table 3 shows unadjusted and adjusted odds ratios for each sexual behaviour in the last year by wealth quintile stratified by sex and urban/rural location. In each group there was a negative association between increasing wealth quintile and transactional sex; aORs 0.92 (0.88–0.97), 0.88 (0.81–0.94), 0.92 (0.85-1.00), and 0.84 (0.74–0.95) for rural women, urban women, rural men, and urban men, respectively.

**Table 3:** Weighted odds ratios of sexual behaviours for increasing wealth (per quintile)

		Odds ratios (95% confidence intervals)		
Outcome	Population	Univariable	Adjusted for country and age	Fully adjusted*
Transactional sex	Rural women	0.94 (0.89–0.99)	0.92 (0.88–0.96)	0.92 (0.88–0.97)
	Urban women	0.84 (0.79–0.90)	0.84 (0.79–0.90)	0.88 (0.81–0.94)
	Rural men	0.95 (0.87–1.02)	0.92 (0.85–0.99)	0.92 (0.85-1.00)
	Urban men	0.84 (0.75–0.94)	0.85 (0.76–0.95)	0.84 (0.74–0.95)
High-risk sex	Rural women	1.09 (1.02–1.17)	1.03 (0.98–1.09)	1.04 (0.98–1.10)
	Urban women	0.91 (0.82–1.01)	0.94 (0.87–1.02)	0.98 (0.90–1.06)
	Rural men	1.14 (1.07–1.21)	1.08 (1.02–1.13)	1.08 (1.02–1.13)
	Urban men	0.86 (0.78–0.95)	0.89 (0.82–0.97)	0.90 (0.83–0.98)
Intergenerational sex	Rural women	0.97 (0.94–1.01)	0.97 (0.94–1.01)	1.04 (1.01–1.08)
	Urban women	0.91 (0.86–0.97)	0.94 (0.89-1.00)	1.01 (0.95–1.07)
	Rural men	1.00 (0.96–1.04)	0.99 (0.95–1.04)	0.99 (0.94–1.04)
	Urban men	0.92 (0.86–0.98)	1.00 (0.92–1.09)	1.03 (0.94–1.12)

* Analyses were adjusted for receipt of food or other economic support, secondary education or higher, having received a paycheck in the previous 12 months, ever having been married, age, and survey country

The associations between wealth quintile and high-risk sex were less clear. Among women in rural areas, there was a positive association between wealth and high-risk sex; OR 1.09 (1.02–1.17). However, this association attenuated somewhat upon adjustment; aOR 1.04 (0.98–1.10). For women in urban areas there was a negative association in unadjusted analyses; OR 0.91 (0.82–1.01) that attenuated after adjustment; aOR 0.98 (0.90–1.06). For men in rural areas there was a positive association between wealth and high-risk sex; aOR 1.08 (1.02–1.13) and there was a negative association in urban areas; aOR 0.90 (0.83–0.98).

For women in rural areas there was a positive association between increasing wealth quintile and intergenerational sex; aOR 1.04 (1.01–1.08). There was no evidence of such an association for women in urban areas; aOR 1.01 (0.95–1.07), men in rural areas; aOR 0.99 (0.94–1.04), or men in urban areas; 1.03 (0.94–1.12).

In sensitivity analyses treating wealth quintile as a categorical variable (supplementary Table 8), the point estimates for the associations between wealth and transactional sex generally followed a linear pattern consistent with the odds ratios when treating wealth quintile as a continuous variable. However, for high-risk sex, the directions of the odds ratios for men and women in urban areas (aORs above 1) differed from when treating wealth quintile as a continuous variable (aORs below 1). For men and women in rural areas, the same direction of ORs was observed (aORs above 1), but the trend did not appear linear, although confidence intervals were wide. For intergenerational sex, there was little evidence of associations with wealth quintile among urban women and men, and rural men, which was also seen in the categorised analysis. For rural women, the association of more intergenerational sex with higher wealth, was mostly reflected through higher odds in the highest wealth quintile.

### Sexual Behaviours and Recent HIV

The unadjusted and adjusted odds ratios of having recently acquired HIV for each sexual behaviour are shown in Table 4, with full model results shown in supplementary Table 7. Testing information was unavailable for 5667 (5.6%) of individuals who were excluded from the analysis with recent HIV as the outcome. For women in both rural and urban areas, the percentage who had recently acquired HIV was higher for those reporting each individual sexual behaviour than those than who did not. In multivariable analyses among women in rural areas, there were positive associations between high-risk sex and recent HIV; aOR 3.40 (1.34–8.59). However, evidence was much weaker for transactional sex; aOR 1.73 (0.71–4.18) and intergenerational sex 1.66 (0.81–3.41). For women in urban areas, there was an association with high-risk sex; aOR 2.86 (1.30–6.32), and also intergenerational sex; aOR 2.60 (1.22–5.58), but not for transactional sex; 1.61 (0.63–4.10). For men in both rural and urban areas there was no evidence of any associations between sexual behaviours and having recently acquired HIV. For women and men in both rural and urban areas there was a positive association between having recently acquired HIV and regional HIV viraemia levels (supplementary Table 9).

**Table 4:** Associations between having recently acquired HIV and sexual behaviours among HIV-negative women and men (weighted)

		Odds ratios (95% confidence intervals)	
	% with recent HIV	Univariable	Multivariable*
**Rural women (N: 31,647, 88 with recent HIV)**			
No transactional sex	0.2%	1	1
Transactional sex	0.4%	2.18 (0.99–4.76)	1.73 (0.71–4.18)
No high-risk sex	0.2%	1	1
High-risk sex	0.8%	4.16 (1.89–9.19)	3.40 (1.34–8.59)
No intergenerational sex	0.2%	1	1
Intergenerational sex	0.3%	1.78 (0.84–3.75)	1.66 (0.81–3.41)
**Urban women (N: 15,698, 63 with recent HIV)**			
No transactional sex	0.2%	1	1
Transactional sex	0.5%	2.05 (0.84-5.00)	1.61 (0.63–4.10)
No high-risk sex	0.2%	1	1
High-risk sex	1.2%	4.89 (2.27–10.53)	2.86 (1.30–6.32)
No intergenerational sex	0.2%	1	1
Intergenerational sex	0.6%	2.74 (1.31–5.75)	2.60 (1.22–5.58)
**Rural men (N: 25,812, 43 with recent HIV)**			
No transactional sex	0.1%	1	1
Transactional sex	0.1%	1.06 (0.24–4.75)	0.97 (0.20–4.66)
No high-risk sex	0.1%	1	1
High-risk sex	0.2%	1.41 (0.43–4.57)	1.39 (0.39–4.98)
No intergenerational sex	0.1%	1	1
Intergenerational sex	0.3%	3.39 (1.46–7.86)	1.84 (0.68–4.99)
**Urban men (N: 11,424, 20 with recent HIV)**			
No transactional sex	0.1%	1	1
Transactional sex	0.0%	0.47 (0.06–3.70)	0.35 (0.04–3.09)
No high-risk sex	0.1%	1	1
High-risk sex	0.4%	6.04 (1.19–30.64)	3.81 (0.85–17.12)
No intergenerational sex	0.1%	1	1
Intergenerational sex	0.2%	3.29 (0.81–13.33)	1.92 (0.47–7.83)

*Adjusted for sexual risk behaviour variables, age, and an interaction between survey country and regional viraemia. Regional viraemia is defined as the percentage of individuals in the enumeration area with unsuppressed HIV-1 viral loads

### Drought and Recent HIV

Unadjusted and adjusted odds ratios for having recently acquired HIV, including exposure to drought, are shown in Table 5 for women and Table 6 for men. For women in rural areas, those exposed to drought had higher odds of having recently acquired HIV (2.26 [95%CI: 1.44–3.56]) in unadjusted analyses, attenuating to 2.10 (95%CI: 1.23–3.60) in analyses adjusted for age and an interaction between survey country and regional viraemia. In analyses also adjusted for demographic variables and sexual behaviours, the association held (2.10 [1.17–3.77]). This fully adjusted association also held when using a more stringent cut-off to define drought (2.46 [1.38–4.42]) and when not using survey weighting (2.20 [1.29–3.74]) (supplementary Table 8). For women in urban areas (0.74 [0.29–1.88]), and men in both rural (0.52 [0.19–1.45]) and urban areas (1.47 [0.40–5.45]), there was no evidence of an association between having been exposed to drought and having recently acquired HIV (Tables 5 and 6). For these groups, there was also no evidence of an association in sensitivity analyses using a more stringent cut-off to define drought and when not using survey weighting (supplementary Table 10). When treating age and wealth quintile as categorical variables, analyses produced similar results when the model was able to converge (supplementary Table 11).

**Table 5:** Adjusted weighted odds ratios of having recently acquired HIV among women*

	Rural			Urban		
	Unadjusted	Adjusted	Further adjusted	Unadjusted	Adjusted	Further adjusted
Drought	2.26 (1.44–3.56)	2.10 (1.23–3.60)	2.10 (1.17–3.77)	1.44 (0.75–2.75)	0.62 (0.25–1.52)	0.74 (0.29–1.88)
Age (per 10-year increase)	1.13 (0.89–1.43)	1.13 (0.90–1.43)	1.08 (0.76–1.54)	1.11 (0.88–1.39)	1.13 (0.90–1.41)	0.81 (0.57–1.16)
Eswatini and regional viraemia interaction	1.16 (1.11–1.21)	1.13 (1.07–1.18)	1.15 (1.09–1.21)	1.05 (0.96–1.16)	1.07 (0.97–1.17)	1.07 (0.97–1.18)
Lesotho and regional viraemia interaction	1.13 (1.08–1.18)	1.09 (1.04–1.14)	1.08 (1.03–1.14)	1.09 (1.04–1.14)	1.11 (1.05–1.18)	1.11 (1.04–1.18)
Tanzania and regional viraemia interaction	1.15 (1.05–1.27)	1.16 (1.06–1.26)	1.18 (1.08–1.28)	0.73 (0.56–0.96)	0.72 (0.54–0.94)	0.72 (0.55–0.94)
Uganda and regional viraemia interaction	1.22 (1.09–1.36)	1.23 (1.11–1.37)	1.20 (1.08–1.34)	1.05 (0.90–1.23)	1.04 (0.89–1.22)	0.98 (0.81–1.19)
Zambia and regional viraemia interaction	1.13 (1.06–1.20)	1.09 (1.02–1.17)	1.09 (1.01–1.17)	1.10 (1.03–1.18)	1.12 (1.05–1.21)	1.14 (1.07–1.22)
Secondary education or higher	1.19 (0.61–2.35)		1.55 (0.67–3.58)	0.72 (0.37–1.37)		0.71 (0.31–1.64)
Received paycheck in last 12 months	1.28 (0.69–2.38)		1.20 (0.58–2.45)	2.44 (1.47–4.05)		2.37 (1.38–4.09)
Ever married	1.89 (0.86–4.18)		1.93 (0.73–5.09)	2.80 (1.43–5.46)		2.66 (1.21–5.88)
No support	1		1	1		1
Food support (vs. no support)	0.64 (0.27–1.51)		0.54 (0.23–1.25)	2.55 (0.92–7.07)		2.83 (1.01–7.91)
Other economic support (vs. no support)	0.57 (0.20–1.67)		0.35 (0.13–0.94)	NA		NA
Severe food insecurity	2.24 (1.03–4.86)		2.14 (0.91–5.03)	1.60 (0.67–4.32)		1.40 (0.56–3.49)
Wealth quintile	0.91 (0.71–1.16)		0.88 (0.68–1.13)	0.84 (0.65–1.08)		0.95 (0.68–1.31)
Transactional sex	2.17 (0.99–4.76)		1.62 (0.64–4.06)	2.05 (0.84-5.00)		1.39 (0.53–3.59)
High-risk sex	4.16 (1.89–9.19)		3.42 (1.38–8.44)	4.89 (2.27–10.53)		2.83 (1.30–6.17)
Intergenerational sex	1.78 (0.84–3.75)		1.57 (0.72–3.39)	2.74 (1.31–5.75)		2.19 (1.02–4.71)

Regional viraemia is defined as the percentage of individuals in the enumeration area with unsuppressed HIV-1 viral loads (the odds ratio is per percentage increase)

*Among women testing negative for HIV antibodies or testing positive with evidence of recent HIV infection

**Table 6:** Adjusted weighted odds ratios of having recently acquired HIV among men*

	Rural			Urban		
	Unadjusted	Adjusted	Further adjusted	Unadjusted	Adjusted	Further adjusted
Drought	0.67 (0.29–1.51)	0.52 (0.20–1.39)	0.52 (0.19–1.45)	1.70 (0.55–5.24)	1.23 (0.37–4.16)	1.47 (0.40–5.45)
Age (per 10-year increase)	1.64 (1.33–2.03)	1.67 (1.35–2.07)	1.17 (0.79–1.72)	1.45 (1.05–2.02)	1.49 (1.06–2.09)	1.01 (0.48–2.09)
Eswatini and regional viraemia interaction	1.08 (0.97–1.20)	1.12 (1.02–1.22)	1.12 (1.00-1.25)	1.17 (1.08–1.27)	1.16 (1.06–1.27)	1.16 (1.05–1.28)
Lesotho and regional viraemia interaction	1.14 (1.08–1.20)	1.18 (1.12–1.23)	1.17 (1.10–1.25)	1.08 (1.01–1.16)	1.07 (1.00-1.14)	1.08 (1.00-1.16)
Tanzania and regional viraemia interaction	1.15 (0.97–1.36)	1.14 (0.96–1.35)	1.14 (0.97–1.34)	0.61 (0.27–1.39)	0.61 (0.27–1.40)	0.65 (0.29–1.45)
Uganda and regional viraemia interaction	1.24 (1.09–1.41)	1.24 (1.09–1.41)	1.21 (1.05–1.39)	1.21 (0.96–1.52)	1.22 (0.97–1.53)	1.18 (0.92–1.51)
Zambia and regional viraemia interaction	1.05 (0.92–1.19)	1.08 (0.95–1.23)	1.08 (0.95–1.23)	1.01 (0.89–1.15)	1.01 (0.89–1.14)	1.01 (0.90–1.14)
Secondary education or higher	0.97 (0.44–2.16)		1.44 (0.63–3.27)	0.81 (0.24–2.74)		0.83 (0.18–3.92)
Received paycheck in last 12 months	2.21 (1.01–4.84)		1.58 (0.74–3.39)	14.41 (3.60-57.75)		9.45 (2.39–37.29)
Ever married	16.03 (4.11–62.49)		10.92 (2.26–52.76)	4.98 (1.25–19.83)		3.38 (0.55–20.87)
No support	1		1	1		1
Food support (vs. no support)	1.15 (0.25–5.35)		1.09 (0.23–5.20)	NA		NA
Other economic support (vs. no support)	4.09 (0.59–28.48)		3.81 (0.52–27.89)	3.37 (0.41–27.63)		2.42 (0.26–22.84)
Severe food insecurity	1.56 (0.56–4.36)		1.54 (0.56–4.23)	1.86 (0.42–8.15)		1.70 (0.39–7.42)
Wealth quintile	0.98 (0.73–1.30)		0.93 (0.70–1.24)	0.94 (0.61–1.46)		0.99 (0.57–1.70)
Transactional sex	1.06 (0.24–4.75)		0.84 (0.18–3.99)	0.47 (0.06–3.70)		0.37 (0.04–3.10)
High-risk sex	1.41 (0.43–4.57)		1.47 (0.41–5.27)	6.04 (1.19–30.64)		3.30 (0.76–14.36)
Intergenerational sex	3.39 (1.46–7.86)		1.71 (0.67–4.36)	3.29 (0.81–13.33)		1.73 (0.47–6.45)

Regional viraemia is defined as the percentage of individuals in the enumeration area with unsuppressed HIV-1 viral loads (the odds ratio is per percentage increase)

*Among men testing negative for HIV antibodies or testing positive with evidence of recent HIV infection

## Discussion

We found that women in rural areas who had recently experienced drought had increased odds of having recently acquired HIV. We also examined the individual links in the hypothesised chain linking drought and HIV. Women and men in rural areas, but not urban areas, that had been exposed to drought had increased odds of being in poverty. Behavioural patterns differed by levels of poverty, with associations identified between poverty and various self-reported recent sexual behaviours. For women and men in both rural and urban areas, there was a negative association between increasing wealth and transactional sex, whilst there was a positive association between increasing wealth and intergenerational sex for women in rural areas. There was weak evidence of a positive association between increasing wealth and high-risk sex among women in rural areas, whilst there was stronger evidence of a positive association among rural men, but the opposite pattern among urban men. Finally, there were increased odds of having recently acquired HIV among women in rural areas reporting high-risk sex, and women in urban areas reporting high-risk sex or intergenerational sex, with no strong associations observed among men. This provides some evidence that drought may be linked to increased HIV transmission risk through intermediary mechanisms as previously hypothesised [1], particularly among women in rural areas. This suggests that as the frequency and severity of droughts in SSA increases, progress towards HIV prevention targets in the region may be hindered.

### Comparison with other Literature

Few analyses have investigated the associations between drought and HIV incidence or prevalence. Burke et al. used Demographic Health Survey (DHS) data to examine the associations between local rainfall shocks and HIV prevalence, finding that infection rates in rural areas where HIV is endemic increase by 11% for every recent drought [16]. Using the Lesotho PHIA survey, Low et al. also found that drought in rural areas was associated with higher HIV prevalence and riskier sexual behaviour in young women [30]. Similarly, Treibich et al’s analysis of Malawi’s DHS data found that experiencing drought increased the likelihood that women employed in agriculture engaged in transactional sex, and that a single drought increased HIV prevalence by around 15% among men and women [31]. Austin et al. used structural equation modelling to demonstrate that droughts are indirect predictors, via factors such as food insecurity, of the percentage of a country’s population living with HIV who are women [18]. Lastly, Epstein et al. used multi-country DHS data to show that experiencing droughts was associated with higher odds of condomless sex and lower odds of HIV testing in the prior year [29].

Studies in SSA have looked at the associations between related variables in the theoretical framework linking drought and HIV [1]. Droughts are associated with extreme poverty in SSA [45], whilst the World Bank has estimated that climate change, including droughts, will lead to millions of people in SSA falling into poverty [46], partially through reliance on subsistence agriculture [47]. The supplementary materials (page 21) contain an in-depth discussion of the literature on the relationships between poverty and sexual risk behaviours and these behaviours and HIV.

### Strengths and Limitations

Strengths of this analysis include the large sample taken from nationally representative bio-behavioural surveys across five countries, and our ability to look at multiple outcomes in the theorised pathway between drought and recent HIV, whilst adjusting for multiple confounders at each step. These results should be generalisable to other similar settings in the region. The biggest limitation is the cross-sectional nature of the surveys, which does not provide direct evidence of the causal relationships in the pathway. However, the period in which the data on rainfall/drought were gathered preceded the surveys, so it is likely that a period of extended drought caused subsequent poverty, although poorer people may have less resources than wealthier counterparts to migrate away from drought-affected areas. Rural areas are perhaps more affected by drought due to the high prevalence of subsistence farming. Unfortunately, we could not adjust for employment type such as farming because the employment questions could only capture formal, rather than casual employment. Instead, we had to use the rural/urban variable as a simple proxy for dominant types of employment. There will also likely be other confounders that we were unable to account for. Having received a pay check in the last 12 months is likely to exclude people who are in casual employment, but, nevertheless, it should act as a marker for more secure employment.

The cross-sectional survey limitation also applies to other outcomes we investigated. To minimise the effect of considering outcome variables that occurred before a predictor, we included sexual behaviours from the last year, whilst the recent HIV infections are likely to have occurred in the few months before the survey took place, although wealth is measured at the time of the survey. Much of the data was self-reported, which could be subject to recall bias, social desirability bias, and some difficulty in defining what could count as transactional sex in more long-term relationships. The sexual behaviour questions were those most affected by missingness, perhaps due to their sensitive nature. This missingness could explain why some of the sexual behaviours were not strongly associated with recent HIV. These various limitations regarding the order that variables may have occurred and the biases around them, may explain why the association between drought and recent HIV among women in rural areas did not attenuate upon adjustment for other variables such as wealth and sexual behaviours. Alternatively, this could be due to not including data on other potential mediating factors such as mental health or gender-based violence, which has been demonstrably under-reported in the PHIA surveys [48].

There could also have been some misclassification of drought severity, however, we believe that our results are reasonably robust due to the use of 2 years of data to define a drought period and using a relative rank rather than an absolute value for our drought indicator, to indicate a departure from normal weather. We also performed a sensitivity analysis using a more stringent cut-off to define drought. Finally, our definition of wealth/poverty was relative to others in that country, rather than absolute (e.g., the $2.15 per day international poverty definition). An absolute measure was unavailable in the data, while purchasing power is usually justified relative to the setting, so the measure should be robust.

### Implications

The links between drought and HIV, highlighted in our study, could have effects that are particularly severe in countries in Southern Africa where the HIV prevalence is high, sometimes > 10% of adults [49], and the frequency and severity of droughts is projected to increase due to climate change [2, 3]. Our results regarding the associations between poverty and HIV are also perhaps applicable to other causes of income shocks, such as the coronavirus disease pandemic [50] or rising food and commodity prices. With countries in SSA accounting for a very small percentage of the global emissions driving global warming, the interventions needed to stop this process would require a global response [2]. Alternatively, more localised responses may be appropriate. There is evidence that social protection cash transfer programs can be effective at reducing risky sexual behaviours, particularly among young women [51]. Additionally, further increasing ART coverage and viral suppression levels among people living with HIV could also help reduce HIV transmission even in the presence of increased risky sexual practices, as persons with undetectable viral loads have been shown to not transmit HIV [52]. However, adherence to ART can decrease in the presence of food insecurity [17, 25], which is strongly correlated to poverty and drought, highlighting the need for interventions to mitigate this problem.

## Conclusions

Our results provide increased evidence of the associations linking drought with increasing HIV transmission among women in rural areas of SSA via poverty and changing sexual behaviours. Interventions that could mitigate this effect of drought on HIV transmission should be considered. Future research should use longitudinal data to elucidate the temporal and causal associations for the pathway linking drought with increased HIV transmission.

### Electronic Supplementary Material

Below is the link to the electronic supplementary material.

Supplementary Material 1

## Data Availability

The PHIA datasets used in these analyses are available upon request on the PHIA website: https://phia-data.icap.columbia.edu/. The data file designed by the World Food Programme’s Vulnerability Analysis and Mapping Geospatial Analysis Team that compares historical rainfall patterns with rainfall between June 2014 to May 2016 is available on request - please contact the corresponding author for access as the datasets are very large.

## Abbreviations

aOR: Adjusted odds ratio
ART: Antiretroviral therapy
CHIRPS: Climate Hazards Group InfraRed Precipitation with Station Data
DBS: Dried blood spots
DHS: Demographic health survey
HIV: Human immunodeficiency virus
OR: Odds ratio
PHIA: Population-Based HIV Impact Assessment
RNA: Ribonucleic acid
SSA: Sub-Saharan Africa
UNAIDS: Joint United Nations Programme on HIV/AIDS

## References

[1] UNAIDS. Climate Change and AIDS: A Joint Working Paper, 2008.

[2] Trisos CH, Adelekan IO, Totin E et al. 2022: Africa. Climate Change 2022: Impacts, Adaption and Vulnerability. Cambridge, UK and New York, NY, USA.: Cambridge University Press,; 2022:1285–455.

[3] NASA. : Earth Observatory. Drought Threatens Millions in Southern Africa, 2019.

[4] Van Loon AF, Gleeson T, Clark J (2016). Drought in the Anthropocene. Nat Geosci.

[5] Morefield PE, Fann N, Grambsch A, Raich W, Weaver CP. Heat-related health impacts under scenarios of Climate and Population Change. Int J Env Res Pub He 2018;15(11). 10.3390/ijerph15112438 PMC6266381 30388822

[6] Campbell-Lendrum D, Manga L, Bagayoko M, Sommerfeld J. Climate change and vector-borne diseases: what are the implications for public health research and policy? Philos T R Soc B 2015;370(1665). 10.1098/rstb.2013.0552 PMC4342958 25688013

[7] Beltrame L, Vineer HR, Walker JG, Morgan ER, Vickerman P, Wagener T. Discovering environmental management opportunities for infectious disease control. Sci Rep-Uk 2021;11(1). 10.1038/s41598-021-85250-1 PMC7979760 33742016

[8] Rebaudet S, Sudre B, Faucher B, Piarroux R (2013). Environmental determinants of Cholera outbreaks in Inland Africa: a systematic review of Main Transmission Foci and Propagation routes. J Infect Dis.

[9] Stanke C, Kerac M, Prudhomme C, Medlock J, Murray V. Health effects of drought: a systematic review of the evidence. PLoS Curr 2013;5. 10.1371/currents.dis.7a2cee9e980f91ad7697b570bcc4b004 PMC3682759 23787891

[10] The Lancet HIV (2020). The syndemic threat of food insecurity and HIV. The Lancet HIV.

[11] Masih I, Maskey S, Mussa FEF, Trambauer P (2014). A review of droughts on the African continent: a geospatial and long-term perspective. Hydrol Earth Syst Sc.

[12] Ngcamu BS, Chari F. Drought influences on Food Insecurity in Africa: a systematic literature review. Int J Environ Res Public Health 2020;17(16). 10.3390/ijerph17165897 PMC7460121 32823825

[13] Krishnan S, Dunbar MS, Minnis AM, Medlin CA, Gerdts CE, Padian NS (2008). Poverty, gender inequities, and women’s risk of human immunodeficiency virus/AIDS. Ann N Y Acad Sci.

[14] McCoy SI, Ralph LJ, Njau PF, Msolla MM, Padian NS (2014). Food Insecurity, Socioeconomic Status, and HIV-Related risk behavior among women in Farming households in Tanzania. Aids Behav.

[15] Weiser SD, Young SL, Cohen CR (2011). Conceptual framework for understanding the bidirectional links between food insecurity and HIV/AIDS. Am J Clin Nutr.

[16] Burke M, Gong E, Jones K (2015). Income shocks and HIV in Africa. Econ J.

[17] Orievulu KS, Ayeb-Karlsson S, Ngema S (2022). Exploring linkages between drought and HIV treatment adherence in Africa: a systematic review. Lancet Planet Health.

[18] Austin KF, Noble MD, Berndt VK (2021). Drying climates and Gendered suffering: Links between Drought, Food Insecurity, and women’s HIV in Less-developed countries. Soc Indic Res.

[19] Wamoyi J, Stobeanau K, Bobrova N, Abramsky T, Watts C. Transactional sex and risk for HIV infection in sub-saharan Africa: a systematic review and meta-analysis. J Int Aids Soc 2016;19. 10.7448/IAS.19.1.20992 PMC5095351 27809960

[20] Bajunirwe F, Semakula D, Izudi J (2020). Risk of HIV infection among adolescent girls and young women in age-disparate relationships in sub-saharan Africa. Aids.

[21] Leclerc-Madlala S (2008). Age-disparate and intergenerational sex in southern Africa: the dynamics of hypervulnerability. Aids.

[22] Pinkerton SD, Abramson PR (1997). Effectiveness of condoms in preventing HIV transmission. Soc Sci Med.

[23] Pascoe SJS, Langhaug LF, Mavhu W et al. Poverty, Food Insufficiency and HIV infection and sexual Behaviour among Young Rural Zimbabwean women. PLoS ONE 2015;10(1). 10.1371/journal.pone.0115290 PMC4307980 25625868

[24] McMichael C (2015). Climate change-related migration and infectious disease. Virulence.

[25] Chop E, Duggaraju A, Malley A (2017). Food insecurity, sexual risk behavior, and adherence to antiretroviral therapy among women living with HIV: a systematic review. Health Care Women in.

[26] Singer AW, Weiser SD, Mccoy SI (2015). Does Food Insecurity Undermine adherence to antiretroviral therapy? A systematic review. Aids Behav.

[27] Nagata JM, Hampshire K, Epstein A et al. Analysis of heavy rainfall in Sub-saharan Africa and HIV Transmission Risk, HIV Prevalence, and sexually transmitted infections, 2005–2017. Jama Netw Open 2022;5(9). 10.1001/jamanetworkopen.2022.30282 PMC9459663 36074468

[28] Baker RE (2020). Climate change drives increase in modeled HIV prevalence. Clim Change.

[29] Epstein A, Nagata JM, Ganson KT et al. Drought, HIV Testing, and HIV Transmission Risk behaviors: a Population-based study in 10 high HIV Prevalence Countries in Sub-saharan Africa. Aids Behav 2022. 10.1007/s10461-022-03820-4 PMC11909628 36066761

[30] Low AJ, Frederix K, McCracken S et al. Association between severe drought and HIV prevention and care behaviors in Lesotho: a population-based survey 2016–2017. Plos Med 2019;16(1). 10.1371/journal.pmed.1002727 PMC6331084 30640916

[31] Treibich C, Bell E, Blanc E, Lepine A. From a drought to HIV: an analysis of the effect of droughts on transactional sex and sexually transmitted infections in Malawi. Ssm-Popul Hlth 2022;19. 10.1016/j.ssmph.2022.101221 PMC9508466 36164494

[32] Muchomba FM, Wang JSH, Agosta LM (2014). Women’s land ownership and risk of HIV infection in Kenya. Soc Sci Med.

[33] Gillies P, Tolley K, Wolstenholme J (1996). Is AIDS a disease of poverty?. AIDS Care.

[34] Magadi MA (2013). The disproportionate high risk of HIV infection among the Urban Poor in Sub-saharan Africa. Aids Behav.

[35] ICAP at Columbia. PHIA Project. 2016. https://phia.icap.columbia.edu/ (accessed 02/09/2022.

[36] Sachathep K, Radin E, Hladik W (2021). Population-based HIV Impact assessments Survey methods, response, and Quality in Zimbabwe, Malawi, and Zambia. Jaids-J Acq Imm Def.

[37] Funk C, Peterson P, Landsfeld M et al. The climate hazards infrared precipitation with stations-a new environmental record for monitoring extremes. Sci Data 2015;2. 10.1038/sdata.2015.66 PMC4672685 26646728

[38] Low A, Gummerson E, Schwitters A et al. Food insecurity and the risk of HIV acquisition: findings from population-based surveys in six sub-saharan African countries (2016–2017). Bmj Open 2022;12(7). 10.1136/bmjopen-2021-058704 PMC9277378 35820770

[39] Stoebenau K, Heise L, Wamoyi J, Bobrova N (2016). Revisiting the understanding of transactional sex in sub-saharan Africa: a review and synthesis of the literature. Soc Sci Med.

[40] Luke N (2003). Age and economic asymmetries in the sexual relationships of adolescent girls in sub-saharan Africa. Stud Family Plann.

[41] Schaefer R, Gregson S, Eaton JW (2017). Age-disparate relationships and HIV incidence in adolescent girls and young women: evidence from Zimbabwe. Aids.

[42] Steffenson AE, Pettifor AE, Seage GR, Rees HV, Cleary PD (2011). Concurrent sexual partnerships and human immunodeficiency Virus Risk among South African Youth. Sex Transm Dis.

[43] Maughan-Brown B, Evans M, George G. Sexual Behaviour of Men and Women within Age-Disparate Partnerships in South Africa: Implications for Young Women’s HIV Risk. PLoS ONE 2016;11(8). 10.1371/journal.pone.0159162 PMC4985138 27526116

[44] PHIA. Population-based HIV Impact Assessment (PHIA) Data Use Manual. New York, NY., 2019.

[45] Azzarri C, Signorelli S. Climate and poverty in Africa South of the Sahara. World Dev 2020;125. 10.1016/j.worlddev.2019.104691 PMC6853414 31902973

[46] Hallegate S, Bangalore M, Bonzanigo L (2016). Shock waves: managing the impacts of Climate Change on Poverty.

[47] Hope KR (2009). Climate change and poverty in Africa. Int J Sust Dev World.

[48] Currie DW, Apondi R, West CA et al. A comparison of two population-based household surveys in Uganda for assessment of violence against youth. PLoS ONE 2021;16(12). 10.1371/journal.pone.0260986 PMC8691642 34932585

[49] UNAIDS, Global HIV. & AIDS statistics - Fact sheet. 2022. https://www.unaids.org/en/resources/fact-sheet (accessed 06/09/2022.

[50] Moyer JD, Verhagen W, Mapes B (2022). How many people is the COVID-19 pandemic pushing into poverty? A long-term forecast to 2050 with alternative scenarios. PLoS ONE.

[51] Stoner MCD, Kilburn K, Godfrey-Faussett P, Ghys P, Pettifor AE (2021). Cash transfers for HIV prevention: a systematic review. Plos Med.

[52] Cohen MS, Chen YQ, McCauley M (2016). Antiretroviral therapy for the Prevention of HIV-1 transmission. N Engl J Med.

