# Supplementary material for: Investigating the Associations between Drought, Poverty, High-Risk Sexual Behaviours, and HIV Incidence in Sub-Saharan Africa: A Cross-Sectional Study
Source: AIDS Behav. 2024 Feb 20;28(5):1752–65. doi: 10.1007/s10461-024-04280-8 (PMC11069459; doi:10.1007/s10461-024-04280-8)
Supplement: Supplementary file 1 — Supplementary Material 1 [file 10461_2024_4280_MOESM1_ESM.docx]

**INVESTIGATING THE ASSOCIATIONS BETWEEN DROUGHT, POVERTY, HIGH-RISK SEXUAL BEHAVIOURS, AND HIV INCIDENCE IN SUB-SAHARAN AFRICA: A CROSS-SECTIONAL STUDY**

**APPENDIX**

**Contents**

Page 2 - PHIA survey design

Page 3 - Supplementary table 1: PHIA survey sampling frames and sample sizes

Page 5 - Supplementary table 2: PHIA survey eligibility criteria

Page 6 - Supplementary table 3: PHIA survey consent procedures

Page 8 - PHIA survey staff training

Page 9 - Supplementary table 4: PHIA survey staff

Page 10 - PHIA survey data collection and cleaning

Page 11 - Supplementary table 5: PHIA survey response rates

Page 13 - Numbers included in analyses and missing data

Page 14 - Climate context preceding and during PHIA data collection

Page 15 - Supplementary figure 1: Climate conditions preceding data collection in each country

Page 16 - Supplementary table 6: Proportion of country affected by drought (defined as SPI-3 < -1) in Oct-Dec 2015 and Oct-Dec 2016

Page 17 - Generation of wealth quintiles in PHIA surveys

Page 18 – Supplementary table 7: Weighted multivariable odds ratios of poverty, with age categorised

Page 19 – Supplementary table 8: Full adjusted weighted odds ratios of sexual behaviours for each wealth quintile

Page 20 - Supplementary table 9: Full adjusted model results for sexual behaviours and recent HIV

Page 21 - Supplementary table 10: Sensitivity analyses for the association between drought and recent HIV

Page 22 – Supplementary table 11: Full adjusted weighted odds ratios of having recently acquired HIV, with age and wealth quintile categorised

Page 23 - In depth comparison with other literature

Page 24 - Supplementary table 12: STrengthening the Reporting of OBservational studies in Epidemiology (STROBE) checklist

Page 27 - References.

**PHIA survey design**

The data-use manual for PHIA surveys is available here:

<https://phia-data.icap.columbia.edu/storage/Country/28-09-2021-22-01-17-615390ad6a147.pdf>

The final reports for each of the five included PHIA surveys are available here below. Each survey report contains details of the sampling frame and design, eligibility criteria, recruitment, consent procedures, survey implementation, testing procedures, response rates, and key findings, as well as the questionnaires.

**Eswatini:** <https://phia.icap.columbia.edu/wp-content/uploads/2020/02/SHIMS2_Final-Report_05.03.2019_forWEB.pdf>

**Lesotho:** <https://phia.icap.columbia.edu/wp-content/uploads/2020/02/LePHIA_FinalReport_Web.pdf>

**Tanzania:** <https://phia.icap.columbia.edu/wp-content/uploads/2020/02/FINAL_THIS-2016-2017_Final-Report__06.21.19_for-web_TS.pdf>

**Uganda:** <https://phia.icap.columbia.edu/wp-content/uploads/2020/02/UPHIA_Final_Report_Revise_07.11.2019_Final_for-web.pdf>

**Zambia:** <https://phia.icap.columbia.edu/wp-content/uploads/2019/03/ZAMPHIA-Final-Report__2.26.19.pdf>

Each survey used a two-stage, stratified cluster sample design. Information from each report is included in the tables below.

**Supplementary table 1:** PHIA survey sampling frames and sample sizes

| **Eswatini** | The sampling frame was comprised of all households in the country based on the 2007 Swaziland Population and Housing Census, which includes 2,064 enumeration areas (EA), containing an estimated 212,195 households. The first stage selected 286 EA (clusters) using a probability proportional to size method. The 286 EAs were stratified by four geographical regions (Hhohho, Manzini, Shiselweni, and Lubombo), with each EA defined by rural and urban status. During the second stage, a sample of households was randomly selected within each EA, or cluster, using an equal probability method, where the average number of households selected per cluster was 20 and the actual number of households selected per cluster ranged from 15 to 43. The sample size was calculated to provide a representative national estimate of HIV incidence among adults aged 15-49 years with a relative standard error less than or equal to 20.0%. Representative regional estimates of VLS prevalence, among HIV-positive adults aged 15-49 years with 95% confidence intervals (CI) with ±10% bounds around the point estimates, were also used. One-half of the households were randomly selected for inclusion of children, which was designed to provide a representative national estimate of pediatric HIV prevalence with a relative standard error less than or equal to 15.0%. The target sample size was 12,042 for adults, and 3,361 for children. |
| --- | --- |
| **Lesotho** | The sampling frame was comprised of all households in the country based on preliminary data from the 2016 Population and Housing Census, which includes 5,684 residential EAs, containing an estimated 568,429 households. The first stage selected 418 EAs (clusters) using a probability proportional to size method. The 418 EAs were stratified by ten districts: Butha Buthe, Leribe, Berea, Maseru, Mafeteng, Mohale’s Hoek, Quthing, Qacha’s Nek, Mokhotlong and Thaba Tseka. During the second stage, a sample of households was randomly selected within each EA, or cluster, using an equal probability method, where the average number of households selected per cluster was 26 and the actual number of households selected per cluster ranged from 15 to 35. Urban areas were defined by the BOS, and were characterized by either high population density, or of a high level of economic activities or infrastructure. Peri-urban areas were defined as areas with moderate population density, or a lesser extent of economic activities or infrastructure. Rural areas were those with only minimal population density or little infrastructure or economic activities. The sample size was calculated to provide representative national estimates of HIV incidence among older adolescent girls and young women aged 15-24 years and adults aged 15-59 years with a relative standard error less than or equal to 30.0%, as well as representative district estimates of VLS prevalence among HIV-positive adults with 95% confidence intervals (CIs) with ±10% bounds around the point estimates. One-half of the households were randomly selected for inclusion of children, which was designed to provide a representative national estimate of pediatric HIV prevalence with a relative standard error less than or equal to 13.0%. The target sample size was 12,698 for adults and 4,222 for children. |
| **Tanzania** | The sampling frame was comprised of all enumeration areas (EAs in the country based on the 2012 Tanzania Population and Housing Census, which included 106,642 EAs, containing an estimated 9,362,758 households. The first stage selected 526 EAs (clusters) using a Probability Proportional to Size (PPS) method. The 526 EAs were stratified by 31 geographical regions. During the second stage, a sample of households was randomly selected within each EA, using a PPS method, where the average number of households to be selected per EA was 30, but the actual number of households selected per EA ranged from 15 to 61. The sample size was calculated to provide a representative national estimate of HIV incidence among adults aged 15 years and older, with a relative standard error less than or equal to 37.0%, as well as representative regional estimates of VLS prevalence among HIV-positive adults aged 15 years and older, with 95% CI with ±10% bounds around the point estimates for high prevalence regions. Based on the selected households, one-third were randomly selected for inclusion of children aged 0-14 years, which was designed to provide a representative national estimate of pediatric HIV prevalence, with a relative standard error less than or equal to 16.2%. Although the survey also measured VLS in children under the age of 15 years, the sample size has not been powered to assure the same level of precision for pediatric VLS. The target sample size was 31,730 for adults aged 15 years and older, and 8,296 for children aged 0-14 years. |
| **Uganda** | The sampling frame comprised all households in the country, based on the 2014 National Housing and Population census, which includes 80,000 EAs, containing an estimated 7,800,000 households. The first stage selected 520 EAs (clusters) using a probability proportional to size method. The EAs were stratified by ten regions: Central 1, Central 2, Kampala, East-Central, Mid-Eastern, North-East, West Nile, Mid-North, Mid-West, and South-West. During the second stage, a sample of households was randomly selected within each EA, or cluster, using an equal probability method. The sample size was calculated to provide a representative national estimate of HIV incidence among adults with a relative standard error (RSE) less than or equal to 30%, as well as representative regional estimates of VLS prevalence among HIV-positive adults with 95% CIs with ±9% or less bounds around the point estimates. Slightly over one-half (59.3%) of the households were randomly selected for inclusion of children, which was designed to provide a representative national estimate of pediatric HIV prevalence with an RSE less than or equal to 20.0%. The sample size was 33,243 for adults, and 10,956 for children. |
| **Zambia** | The first stage selected 511 enumeration areas (EAs) from the 2010 Census of Population and Housing in Zambia, using a probability proportional to size method. The second stage randomly selected a sample of households in each EA (cluster) using an equal probability method, where the average number of households selected per cluster was 27 and the actual number of households selected per cluster ranged from 11 to 48. The sampling frame for the second stage was defined based on a household listing exercise conducted from August to September 2015, prior to the initiation of data collection. The sample size of selected households was calculated to provide a representative national estimate of HIV incidence among adults aged 15-59 years with a relative standard error less than or equal to 31.2%, as well as representative provincial estimates of VLS prevalence among HIV-positive adults aged 15-59 years with 95% confidence intervals (CIs) ±11.0%. One-half of households were randomly selected for inclusion of children aged 0-14 years, which was designed to provide a representative national estimate of pediatric HIV prevalence with a relative standard error ≤14.6%. The target sample size was 19,168 for adults aged 15-59 years, and 8,974 for children aged 0-14 years. |

**Supplementary table 2:** PHIA survey eligibility criteria

| **Eswatini** | The eligible survey population included: Women and men aged 18 years and older living in the selected households and visitors who slept in the household the night before the survey, who were willing and able to provide written consent in either siSwati or English. Adolescents aged 10-17 years living in the selected households, and visitors who slept in the household the night before the survey, who were willing and able to provide written assent in either siSwati or English, and whose parents or guardians were willing and able to provide written permission for their participation in either siSwati or English. Children aged 0-9 years living in the selected households, and child visitors who slept in the household the night before the survey, whose parents or guardians were willing and able to provide written consent for their participation in either siSwati or English |
| --- | --- |
| **Lesotho** | The eligible survey population included individuals living in the selected households, or visitors who slept in the household the night before the survey who were: ▪ Women and men aged 18-59 years who were willing and able to provide written consent, ▪ Adolescents aged 12-17 who were willing and able to provide written assent, and whose parents or guardians were willing and able to provide written permission for their participation, and ▪ Children aged 0-11 years whose parents or guardians were willing and able to provide written consent for their participation. |
| **Tanzania** | The eligible survey population included: ▪ Females and males aged 18 years and older who slept in the household the night before the survey and were willing and able to provide verbal informed consent in Kiswahili or English. ▪ Persons aged 10-17 years who slept in the household the night before the survey, who were willing and able to provide verbal assent in Kiswahili or English, and whose parents or guardians were willing and able to provide verbal permission for their participation in Kiswahili or English. ▪ Children aged 0-9 years who slept in the household the night before the survey, whose parents or guardians were willing and able to provide verbal informed consent in Kiswahili or English for their participation. |
| **Uganda** | The eligible survey population included: ▪ Women and men aged 18-64 years living in the selected households (and visitors who slept in the household the night before the survey) who were willing and able to provide verbal consent. ▪ Children and adolescents aged 8-17 years living in the selected households (and visitors aged 8-17 years who slept in the household the night before the survey) who were willing and able to provide verbal assent, and whose parents or guardians were willing and able to provide verbal permission for their participation. ▪ Children aged 0-7 years living in the selected households (and visitors aged 0-7 years who slept in the household the night before the survey) whose parents or guardians were willing and able to provide verbal consent for their participation. |
| **Zambia** | Participants were eligible if they were literate in one of the survey languages or could provide a literate witness and were willing and cognitively able to provide consent. The eligible survey population included: ▪ Children aged 0-9 years living in residential households, and child visitors who slept in the household the night before the survey, whose parents or guardians were able to provide consent for their participation. ▪ Young people aged 10-17 years living in residential households, and young visitors who slept in the household the night before the survey, willing and able to provide assent and whose parents or guardians provided permission. ▪ Women and men aged 18-59 years living in residential households, and visitors who slept in the household the night before the survey, who were able to provide consent in one of the eight survey languages (English, Bemba, Nyanja, Lozi, Tonga, Lunda, Luvale, or Kaonde). |

**Supplementary table 3:** PHIA survey consent procedures.

| **Eswatini** | An electronic informed consent form was administered using a tablet computer. At each stage of the consent process, consent was indicated by signing or making a mark on the consent form on the tablet and on a printed copy, which was retained by the participant. A designated head of household provided written consent for household members to participate in the survey, after which individual members were rostered during a household interview. Adults and children with special circumstances (e.g., emancipated minors) provided written consent on the tablet for an interview. After completing the interview, they provided written consent for participation in the biomarker component of the survey, including home-based testing and counselling (HBTC), with return of HIV-testing results and CD4 counts during the household visit. Receipt of tests results was a requirement for participation in the biomarker component. If an individual did not want to receive his or her HIV test result, this was considered a refusal for the biomarker component. Adults were also asked for written consent to store their blood samples in a repository to perform additional tests in the future. Participants aged 10-17 years were asked for assent to the interview and biomarker components after permission was granted by their parents or guardians. Parents provided consent for biomarker testing for minors below the age of assent (ages 0-9 years). Procedures with illiterate participants or participants with a sight disability involved the use of an impartial witness, chosen by the potential participant, who also signed or made a mark on the consent form on the tablet and the printed copy. If no witness could be identified, the potential participant or household (if the head of household was sight disabled or illiterate) was deemed ineligible. |
| --- | --- |
| **Lesotho** | An electronic informed consent form was administered using a tablet. At each stage of the consent process, consent was indicated by signing or making a mark on the consent form on the tablet and on a printed copy, which was retained by the participant. A household-designated head provided written consent for household members to participate in the survey, after which individual members were rostered during a household interview. Adults and emancipated minors then provided written consent on the tablet for an interview. After completing the interview, they provided written consent for participation in the biomarker component of the survey, including HBTC, with return of HIV test results and CD4 counts during the household visit. Receipt of HIV test results was a requirement for participation in the biomarker component. If an individual did not want to receive his or her HIV test result, this was considered a refusal and the survey was concluded. Adults were also asked for written consent to store their blood samples in a repository to perform additional tests in the future, and for consent to share their contact information with the MOH or an implementing partner if HIV positive for active linkage to care. Adolescents aged 12-17 years were asked for assent to the interview and biomarker components after permission was granted by their parents or guardians. Parents provided consent for interview and biomarker testing for children aged 10-11 years, and provided consent for biomarker testing for children below the age of 10 years. In both cases, if a parent or guardian did not want to receive his or her child’s HIV test result, this was considered a refusal and the survey was concluded. Adolescents aged 12-17 years were also asked for consent for linkage to care as in adults, and parents provided consent for children aged 0-11 years. Procedures with illiterate participants, or participants with a sight disability, involved the use of an impartial witness, chosen by the potential participant, who also signed or made a mark on the consent form on the tablet and the printed copy. If no witness could be identified, the potential participant or household (if the head of household was sight disabled or illiterate) was deemed ineligible. |
| **Tanzania** | An electronic informed consent form was administered using a tablet computer. Respondents provided verbal informed consent, which was then noted in the tablet. A designated head of household provided consent for household members to participate in the survey, after which individual members had to provide consent/assent for an interview. After completing the interview, individuals aged 18 years and older provided verbal consent for participation in the biomarker component of the survey, including HBTC and syphilis testing, with return of HIV and syphilis test results and CD4 counts during the household visit. People in the household aged 10-17 years were asked for assent to the interview and biomarker components after permission was granted by their parents or guardians. Parents provided consent for biomarker testing for minors below the age of assent (ages 0-9 years). A CD4 count was conducted for all participants who tested HIV positive and 2.0% of the HIV-negative participants who were randomly selected by a computerized algorithm. Receipt of test results was a requirement for participation in the biomarker component. If an individual did not want to receive his or her HIV test result, this was considered a refusal and the survey was concluded. Participants were also asked for consent/assent to store their blood samples in a repository for future use to perform additional tests. All participants were assessed for cognitive ability prior to obtaining their consent. Cognitive ability to consent was assessed by providing information on survey participation and then asking participants to summarize their understanding of the purpose of the survey and what is requested from them if they chose to participate. In addition to verbal consent, participants who consented were provided with a hardcopy version of the same consent form. |
| **Uganda** | An electronic informed consent form was administered using a tablet. A designated head of household provided consent for household members to participate in the survey, after which individual members were rostered during a household interview. Adults and emancipated minors then provided consent for an interview. After completing the interview, they provided consent for participation in the biomarker component of the survey, including HBTC, with return of HIV test results and CD4 counts during the household visit. Receipt of test results was a requirement for participation in the biomarker component. If an individual did not want to receive his or her HIV test result, this was considered a refusal and the survey was concluded. Adults were also asked for consent to store their blood samples in a repository to perform additional tests in the future. Children and adolescents aged 8-17 years were asked for assent to the interview (ages 15-17 years only) and biomarker components (ages 8-17 years) after permission was granted by their parents or guardians. Parents provided consent for biomarker testing for children below the age of assent (ages 0-7 years). If a parent or guardian did not want to receive their child’s HIV test result, this was considered a refusal and the survey was concluded. Procedures with illiterate participants or participants with a sight disability involved the use of an impartial witness, chosen by the potential participant, who also signed or made a mark on the consent form on the tablet and the printed copy. If no witness could be identified, the potential participant or household (if the head of household was hearing or sight disabled or illiterate) was deemed ineligible. |
| **Zambia** | A designated head of household provided written consent for household members to participate in the survey on an electronic informed consent form administered using a tablet. Individual members were then rostered during a household interview. Persons aged 15-59 (note that parental permission and participant assent were obtained for adolescents 15 to 17 years of age) and emancipated minors (minors 15-17 years of age who are married, have children and or living without parental/guardian supervision) then provided written consent for an interview on the tablet. After completing the interview, they provided written consent for participation in the biomarker component of the survey, which included home-based testing and counselling (HBTC) for HIV, hepatitis B, and syphilis (for persons 15-59 years of age) with return of HIV test results and CD4 count results for those who tested HIV positive, during the household visit. If an individual did not want to receive his or her HIV test result, it was considered a refusal and the survey was stopped. Adults were also asked for written consent for their blood samples to be stored in a repository for future testing. At each stage of the consent process, consent was indicated by signing or making a mark on the consent form on the tablet and on a printed copy, which was retained by the participant. Adolescents aged 10-14 years were asked for assent to the interview and biomarker components after permission was granted by their parents or guardians. Parents or guardians provided consent directly for minors below the age of assent (ages 0-9 years). In both cases, if a parent or guardian did not want to receive his or her HIV test result, this was considered a refusal and the survey was concluded. Procedures with non-literate participants, or participants with a sight disability, involved the use of an impartial witness, chosen by the potential participant who also signed or made a mark on the consent form on the tablet and the printed copy. If no witness could be identified, the potential participant or household (if the head of household was illiterate) was deemed ineligible. |

**PHIA survey staff training**

In each survey, survey staff received training on both the contents of the data collection instruments and tablet use. The training curriculum included:

▪ Scientific objectives of the survey

▪ Survey design and methodology

▪ Completion of survey forms

▪ Data collection

▪ Staff responsibilities

▪ Recruitment of participants

▪ Informed consent procedures including human participants’ protection, privacy, and confidentiality

▪ Blood collection for children and adults, including venipuncture and finger/heel stick

▪ HIV HBTC

▪ CD4 count measurement using a POC PIMA Analyzer

▪ Referral of participants to health and social services

▪ Management and transportation of blood specimens

▪ Biosafety

▪ Communication skills

▪ Protocol deviations, adverse events, and reporting of events

Laboratory staff were trained in specimen management, including sample processing, labeling, and QA. Central laboratory staff were trained in VL measurement, EID, HIV confirmatory testing, and HIV recency testing using the limiting antigen (LAg) avidity enzyme immunoassay (EIA).

**Supplementary table 4:** PHIA survey staff

| **Eswatini** | Fieldwork started on August 30, 2016 and completed on March 31, 2017. Fieldwork was conducted by 16 locally hired field teams composed of a supervisor, six health workers, and a driver. Field teams included both male and female staff and members spoke both siSwati and English. A total of 163 regional coordinators, team supervisors, field health workers, community-mobilization coordinators, and drivers participated in data collection. Survey personnel were selected based on their qualifications and areas of expertise. The field health workers obtained consent, administered the interview, conducted phlebotomy, performed CD4 counts using a point-of-care instrument, and delivered HIV testing services (HTS) for adults and children. The field teams were managed by four regional coordinators and supervised by sixteen team supervisors, who guided and oversaw data collection activities, performed quality checks, and provided technical support. In addition, 15 laboratory technicians processed samples and performed additional procedures for HIV-1 VL, infant virologic HIV testing, and quality control (QC) and QA. National and international monitors periodically conducted direct observation of data collection activities in the field and in the laboratories to provide technical support and ensure quality. |
| --- | --- |
| **Lesotho** | Fieldwork started at the end of November 2016 and was completed in May 2017. Fieldwork was conducted by 24 locally hired field teams composed of a team leader, four nurse interviewers, two additional interviewers, and a driver. A total of 256 field staff including field coordinators, team leaders, nurses, interviewers, community-mobilization coordinators and community mobilizers, and drivers participated in data collection. Survey personnel were selected based on their qualifications and areas of expertise. Each field team was supervised by a team leader, and all teams were overseen by four regional coordinators, who guided and oversaw data collection activities, performed quality checks, and provided technical support. In addition, seven laboratory technicians processed samples and performed additional procedures for HIV-1 VL, infant virological HIV testing, and quality control (QC) and QA. National and international monitors periodically conducted direct observation of data collection activities in the field and in the laboratories to provide technical support and ensure quality. |
| **Tanzania** | A total of 323 participants participated in the training that comprised 187 interviewers, 31 supervisors, 51 facilitators, and 46 laboratory technicians. Facilitators and supervisors were from the NBS, OCGS, ICAP at Columbia University, TACAIDS, NHLQATC, MOH Zanzibar, MoHCDGEC, ZIHHTLP, CDC both Tanzania and Atlanta, U.S., Westat and other staff from the private sector. A total of 46 laboratory technologists and 187 nurse counsellors were recruited from public health facilities after they were identified through a competitive process overseen by the MOH through the Regional Medical Offices. Those who qualified were recruited to serve as interviewers, counsellors, and testers during the survey. The selection criteria for a nurse interviewer for THIS was the individual had to be a certified nurse and accredited by the MOH as an HIV counsellor. Additional survey management, supervision, and support staff were also recruited and trained accordingly prior to data collection. |
| **Uganda** | Fieldwork was conducted by 31 field teams composed of a team leader, two interviewers, two HBTC providers, two lab technicians, and a driver. A total of 256 people, including field coordinators, team leaders, field health workers, HBTC providers, lab technicians, community-mobilization coordinators, and drivers participated in data collection. Survey personnel were selected based on their qualifications and areas of expertise. The interviewers had primary responsibility for obtaining consent and administering the interview. The lab technicians conducted phlebotomy, HIV testing and performed CD4 counts using a point of care (POC) instrument. The HBTC providers were responsible for delivering adult and pediatric counseling and return of test results. The field teams were supervised by four field supervisors and managed by four field coordinators, who guided and oversaw data collection activities, performed quality checks, and provided technical support. In addition, 48 laboratory technicians processed samples at the 10 satellite labs and performed additional procedures centrally for HIV-1 VL, early infant diagnosis (EID), quality control (QC) and quality assurance (QA). National and international monitors periodically conducted direct supervision and observation of data collection activities in the field and in the laboratories in order to provide technical support and ensure quality. |
| **Zambia** | Field work started on March 1, 2016 and completed by August 31, 2016. A total of 47 field teams conducted the survey; however, at any given time, there were a maximum of 37 teams in the field. Each team was composed of one team leader, two interviewers, two nurse counselors, one midwife, and one laboratory technician. Survey personnel were selected based on local language proficiency and profession. While all survey personnel had the responsibility of obtaining consent and administering the interview, nurse counselors, midwives, and laboratory technicians also conducted phlebotomy and other duties as needed. In addition, nurse counselors and midwives provided adult and pediatric HIV counselling. Over 40 laboratory staff processed samples and performed additional tests for HIV-1 viral load, infant virological testing, and quality control (QC) and QA. Teams were supervised by three or four provincial supervisors at a time, along with 40 rotating field monitors who performed spot-checks of teams in the field, providing technical support and assistance where needed. National and international monitors routinely provided direct observation of field work and QA. |

**PHIA survey data collection and cleaning**

For each survey, field data were collected on tablet computers, transmitted to a central server using a secure virtual private network, and stored in a secure PostgreSQL database. Data cleaning was conducted using SAS 9.4 (SAS Institute Inc. Cary, North Carolina, U.S.). Laboratory data were cleaned and merged with the final questionnaire database using unique specimen bar codes and study IDs.

**Supplementary table 5:** PHIA survey response rates

| **Eswatini** | Of the 6,417 selected households, 6,056 and 5,185 were occupied and interviewed, respectively. The overall household response rate (unweighted) was 84.9% (79.3% in urban areas and 87.2% in rural areas). After adjusting for differential sampling probabilities and nonresponse, the overall weighted household response rate was 84.5% (79.1% in urban areas and 87.1% in rural areas). A total of 12,857 adults (5,648 males and 7,209 females) were eligible to participate in the survey. Interview response rates (unweighted) were 86.8% for males and 93.9% for females. Among those adults who were interviewed, 91.9% of males and 95.0% of females (unweighted) also had their blood drawn. Children in half of the selected households were eligible for blood draw. Of the 2,697 eligible children aged 0-9 years, 82.7% of males and 81.8% of females (unweighted) had their blood drawn. Of the 1,300 eligible children aged 10-14 years, interview response rates (unweighted) were 88.9% of males and 92.5% of females. Among 10-14-year-olds interviewed, 98.3% of males and 97.5% of females (unweighted) had their blood drawn. |
| --- | --- |
| **Lesotho** | Of the 10,892 selected households, 9,403 and 8,824 were occupied and interviewed, respectively. The overall household RR (unweighted) was 93.2% (91.2% and 91.4% in urban and peri-urban areas, respectively, and 94.9% in rural areas). After adjusting for differential sampling probabilities and nonresponse, the overall weighted household RR was 93.0%. A total of 14,028 adults (6,135 men and 7,893 women) were eligible to participate in the survey. A total of 12,887 adults participated in the individual interview: interview RR (weighted) were 86.8% for men and 95.1% for women. Among those adults who were interviewed, 88.2% of men and 91.3% of women also had their blood drawn (weighted). In LePHIA, children in half of the selected households were eligible for blood draw. Of the 3,118 eligible children aged 0-9 years, 77.6% of boys and 78.7% of girls had their blood drawn (weighted). Of the 1,752 eligible young adolescents aged 10-14 years, 88.1% of young adolescent boys and 89.7% of young adolescent girls completed interviews and of those, 94.1% of young adolescent boys and 94.8% of young adolescent girls had their blood drawn (weighted). |
| **Tanzania** | Of the 16,198 selected households, 15,504 and 14,811 were occupied and interviewed, respectively. The overall household RR was 94.8%: 95.3% in urban areas and 94.5% in rural areas. Household RR were almost similar in Zanzibar (94.1%) and Tanzania mainland (94.8%). A total of 16,235 males and 19,852 females aged 15 years and older were eligible to participate in THIS. Interview RR were 88.5% for males and 93.2% for females aged 15 years and older. For males and females aged 15 and older, more than nine out of 10 (94.4% and 95.2%, respectively) persons who were interviewed also had their blood drawn. In THIS, children aged 0-14 years in one-third of the selected households were eligible for blood draw. For young persons aged 10-14 years, the interview RR were 91.6% and 92.5% for males and females, respectively. The blood draw RR was 97.1% for males and 97.7% for females. More than nine out of 10 (91.4% for males and 91.6% for females) eligible persons aged 0-9 years had their blood drawn. Blood draw RR among ages 0-9 years were similar between genders in rural areas (92.2%). In urban areas, however, females had a slightly higher RR (89.8%) than males (89.3%). |
| **Uganda** | Of the 13,435 households selected for this survey, 12,812 were occupied, and 12,386 (96.5 %) of these completed a household interview. Overall, the household RR (unweighted) was 96.5% (95.6% for urban and 96.9% for rural areas). After adjusting for differential sampling probability and nonresponse, the overall weighted household RR was 96.7%. A total of 30,581 adults (13,364 men and 17,217 women) were eligible to participate in the survey. A total of 29,383 adults participated in the individual interview: interview RRs were 94.0% for men and 97.9% for women. Among adults who were interviewed, 98.5% of men and 99.0% of women also had their blood drawn. The total RR was 92.4% among men and 96.8% among women. In UPHIA, children in half of the selected households were eligible for blood draw. Of the 10,026 eligible children aged 0-12 years, 96.2% of boys and 96.7% of girls had their blood drawn. Of the 767 eligible adolescents aged 13-14 years, 99.2% of the boys and 99.4% of the girls had their blood drawn. |
| **Zambia** | Of the 13,441 households selected for this survey, 12,193 were occupied, and 89.4% of these completed a household interview. The urban and rural weighted household response rates were almost exactly the same at 89.5% and 89.2%, respectively. Of the 13,317 eligible women and 11,346 eligible men aged 15-59 years, 90.8% of women and 80.4% of men were interviewed. The response rate for biomarker testing among those who completed an individual interview was 88.5% for males and 90.3% for females. Of the 3,593 eligible children aged 10-14 years, 74.6% of males and 79.1% of females completed an interview, while similar percentages of males (90.9%) and females (90.6%) completed biomarker testing. Of 8,053 eligible children aged 0-9 years, about two-thirds (66.9% for males and 66.7% for females) had their blood drawn. |

**Numbers included in analyses and missing data**

Across the five country surveys, response rates varied from 84.5% in Eswatini (N=11673), 89.4% in Zambia (N=21280), 93.0% in Lesotho (N=12887), 94.8% in Tanzania (N=33004), to 96.7% in Uganda (N=29383). We excluded persons aged ≥60 years; 1437 in Eswatini, 3361 in Tanzania, and 997 in Uganda.

For each of the demographic/economic variables, missingness was <1% for each country. Missingness was higher for the sexual behaviour questions. In each survey, some people did not answer the questions about having partners in the previous 12 months and were then subsequently not asked about other sexual behaviours; 175 in Eswatini, 237 in Lesotho, 814 in Tanzania, 188 in Uganda, and 1164 in Zambia. Of those asked the subsequent questions, the variables with >1% missingness varied between countries:

- Eswatini: No relevant questions with >1% missingness.
- Lesotho: Sexual partner ages (N=335) and having ever sold sex (N=521).
- Tanzania: Having sold sex in the past 12 months (N=538).
- Uganda: Sexual partner ages (N=590)
- Zambia: Having ever sold sex (N=1857)

HIV testing information was unavailable for 5667 (5.6%) persons, however, this is accounted for in the weighting.

We also excluded persons with missing data on wealth quintile or education level; 15 in Eswatini, 42 in Lesotho, 22 in Tanzania, 159 in Uganda, and 113 in Zambia.

Therefore, included across the five surveys were 102,081 respondents aged 15-59; 12,845 from Lesotho, 29,621 from Tanzania, 28,227 from Uganda, 10,221 from Eswatini, and 21,167 from Zambia.

# Climate context preceding and during PHIA data collection*

*This section has been taken from *“Food insecurity and the risk of HIV acquisition: findings from population-based surveys in six sub-Saharan African countries (2016-2017)”* by Low et al.(1) and amended, with the permission of the authors.

Analysis carried out by the World Food Programme (WFP) on rainfall and temperature patterns in the past 40 years for several countries in the African continent show that while there are marked temperature increases across the region, the case is less clear cut for rainfall. Of the countries under analysis, Uganda, Tanzania and Zambia show positive rainfall trends over the past 40 years, while negative trends are apparent in eSwatini and Lesotho.

The key rainfall feature for agricultural production and consequently rural food production and food insecurity is the inter-annual (year-on-year) variability in rainfall. In the long run, rainfall variability is a major determinant of livelihoods in the semi-arid tropics as beyond a certain value, purely agriculture-based livelihoods become unfeasible, and households switch progressively to livestock-based livelihoods. These fluctuations subject households to the twin hazards of drought and flood. It is a long-term driver of chronic food insecurity as large and unpredictable year-on-year fluctuations in rainfall amounts prevent households from diversifying the crops they plant and lead them to become more risk averse and conservative in terms of their production strategies. In general, the magnitude of inter-annual variations is much larger than any changes arising from a possible long-term trend.

Another mode of variation in seasonal rainfall that may be present is associated with multi-year periods of drier or wetter than average conditions with inter-annual variability super-imposed on these lower frequency cycles. Of the countries in the study, eSwatini and Lesotho were undergoing drier than average conditions, while both Tanzania and Uganda are in a wetter than average phase. While changes in mean and extreme rainfall have been the object of intense study, rainfall variability has received much less attention. Recent studies indicate that in response to global warming, rainfall variability in tropical areas is expected to increase more than mean precipitation due to greater increase in rainfall extremes(2).

The surveys in this analysis were mostly carried out during a fairly unique period from a climate point of view. From late-2014 to mid-2016 one of the longest and most intense El Niño events on record developed. For Southern Africa in particular, it led to two consecutive droughts. The first season (2014-2015) had a milder impact given suitable regional cereal stocks but already enhanced the food insecurity of the most vulnerable populations. The second season (2015-2016) had major impacts on crop production at regional scale and led to a large spike in food insecurity across the region. While these are separate seasons, the impacts of rainfall deficits in a first season influence the response of crops in the following season, by severely decreasing soil moisture stores and leading to delayed planting in the following season. Hence it made more sense to consider the two seasons together when assessing the extent of the drought. Consecutive droughts have compounding effects on food insecurity – the first year of drought, deplete national and regional stocks and directly impact on households, enhancing their vulnerability due to reducing household savings and increasing sales of productive assets. This enhances the impact of the second year of drought through severe reductions in staple food availability and extreme market prices. This period was followed by two La Niña events in 2016-2017 and 2017-2018, which led to drier than average conditions in East Africa and wetter conditions in Southern Africa.

**Supplementary figure 1: Climate conditions preceding data collection in each country***

**
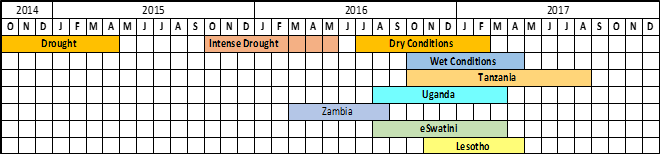
**

*Cells with country names represent months of survey data collection, whereas those cells above the bolded line represent the average conditions in Southern Africa at those times.

**Supplementary table 6:** Proportion of country affected by drought (defined as SPI-3 < -1) in Oct-Dec 2015 and Oct-Dec 2016

| **Country** | **Oct-Dec 2015** | **Oct-Dec 2016** |
| --- | --- | --- |
| Zambia | 15.7% |  |
| eSwatini | 87.3% |  |
| Lesotho | 100.0% |  |
| Uganda |  | 31.8% |
| Tanzania |  | 62.5% |

SPI: Standardised Precipitation Index

**Generation of wealth quintiles in PHIA surveys***

*Extract taken from *Population-based HIV Impact Assessment (PHIA) Data Use Manual. New York, NY. July 2019.(3)*

**Rationale.**

Wealth index methods using survey data on household assets, materials and durable goods have become an established measure of socioeconomic status since their adoption by the Demographic and Health Surveys Program (DHS)(4, 5). These wealth measures are widely considered to be a more accurate construct than income to quantify socioeconomic status in resource-limited settings and are easily discernible via survey questionnaire. DHS has provided commonly accepted guidelines for wealth index construction(5). Wealth index variables (continuous scores and quintiles) have been constructed for analysis using an easily reproducible method across PHIA surveys. Household dwelling characteristic and asset variables used to construct wealth indices vary by PHIA survey and are noted in each survey’s Data Use Manual Supplement. In PHIA datasets, two wealth index variables have been provided: a continuous score (wealthscorecont) and categorical wealth quintile (wealthquintile).

**Method.**

To construct wealth quintiles via DHS methods, the following steps are used:

1. Recode asset variables. Household data include categorical variables about household characteristics, such as construction materials for walls, floors and roof of the household dwelling, source of water, availability of electricity and type of sanitation facilities used, and binary variables indicating ownership of durable goods such as beds, vehicles, and livestock, etc. The specific assets and question wording vary across PHIA surveys (see each PHIA survey’s Data Use Manual Supplement and Survey Questionnaire). Categorical variables are recoded as binary indicator variables (e.g., one variable was created for each floor type and a household receives 1 for the variable indicating their floor type and 0s for all others). Binary variables are coded as 1 (yes) or 0 (no). Generally, missing data are treated as the absence of that asset, and households that do not have data on any assets are not assigned wealth index scores or wealth quintiles.

2. Select the asset variables for inclusion. Asset variables are analyzed using PCA, which is a statistical technique that transforms a number of (correlated) variables into uncorrelated components that capture variability (information) in decreasing order; thus, PCA is a useful dimension reduction technique. DHS recommends using the first component of the model as a summary indicator for wealth (the wealth index). Since assets may vary in relevance in urban and rural settings, PCAs are run separately for urban and rural households, and then for all households combined. Decisions to include or exclude asset variables from either setting may be made on the basis of contextual PHIA Data Use Manual v4.7 - July 2019 38 knowledge; for parsimony, all asset variables that have any variability are included in each analysis.

3. Run PCA and combine results. Three PCAs are run: a “common” model across all households, and models restricted to “urban” and “rural” households. As per convention, the first factor from each model is extracted to obtain three separate wealth indices. The common model wealth index is regressed separately on the urban or rural wealth index for households in those areas, and this regression model is then used to convert each household’s (rural or urban) wealth index into a final “composite” wealth index (wealthscorecont).

4. Generate wealth quintiles. Households are classified into quintiles (wealthquintile) using the composite wealth index. In order to account for the complex survey design, the weighted cumulative distribution of the wealth index is used to identify weighted quintile cut-points. Weights represent the normalized household sampling weight (hhwt0 divided by the mean hhwt0 across all households).

Caveats and other considerations. Wealth indices and quintiles derived using this methodology, are intended to represent relative measures of wealth as compared to other households in the same country. It is important to note that the underlying Principal Components model simply finds the factors that best capture the variation in the data, and does not guarantee a straightforward interpretation. On average, households in higher wealth quintiles should be more wealthy, but there is considerable uncertainty due to limitations of the available asset data and modeling procedure. Wealth is a complex concept that cannot be captured fully in the model, thus wealth indices should be treated as approximate estimates rather than precise measures.

The value of the wealth index should not be thought of as directly proportional to household wealth, or as being measured along a standard baseline that can be compared between different countries or sub-populations. Relative measures should not be applied to subsets of the population; doing so implicitly assumes that the relative distribution of wealth is similar between the total and subsetted population.

For simplicity and to facilitate replication, variables were not selected differently in urban and rural models on the basis of contextual or subjective knowledge. However, this may not be valid if assets are differentially related to wealth across contexts. Sensitivity analyses excluding variables considered to be context-specific (e.g. livestock) or which scored the most differently in the rural and urban models have typically shown that wealth indices are not sensitive to model specification. Alternative socioeconomic indicators are available and the merits of these alternatives are the subject of ongoing debate(6, 7).

**Supplementary table 7:** Weighted multivariable odds ratios of poverty, with age categorised

| **Women** | **Rural** | **Urban** |  |
| --- | --- | --- | --- |
| Drought | 1.28 (1.02-1.61) | 1.24 (0.68-2.27) |  |
| Age 15-19 | 1 | 1 |  |
| Age 20-24 | 0.84 (0.75-0.93) | 0.76 (0.56-1.02) |  |
| Age 25-29 | 0.69 (0.60-0.78) | 0.68 (0.48-0.95) |  |
| Age 30-34 | 0.60 (0.52-0.69) | 0.66 (0.44-0.99) |  |
| Age 35-39 | 0.56 (0.48-0.66) | 0.77 (0.53-1.11) |  |
| Age 40-44 | 0.57 (0.49-0.65) | 0.98 (0.68-1.41) |  |
| Age 45-49 | 0.55 (0.47-0.65) | 0.71 (0.46-1.08) |  |
| Age 50-54 | 0.52 (0.43-0.62) | 0.95 (0.64-1.41) |  |
| Age 55-59 | 0.59 (0.49-0.71) | 0.68 (0.44-1.04) |  |
| Secondary education or higher | 0.25 (0.23-0.28) | 0.15 (0.11-0.20) |  |
| Ever married | 1.53 (1.36-1.74) | 1.40 (1.07-1.84) |  |
| Received paycheck in last 12 months | 0.83 (0.77-0.90) | 0.75 (0.62-0.91) |  |
| Food support (vs no support) | 1.96 (1.32-2.91) | 0.83 (0.39-1.77) |  |
| Other economic support (vs no support) | 1.69 (1.41-2.04) | 2.90 (1.99-4.23) |  |
| Eswatini | 1 | 1 |  |
| Lesotho | 1.13 (0.92-1.39) | 0.12 (0.07-0.20) |  |
| Tanzania | 0.97 (0.75-1.26) | 0.08 (0.05-0.15) |  |
| Uganda | 0.78 (0.60-1.01) | 0.32 (0.18-0.55) |  |
| Zambia | 1.14 (0.93-1.40) | 0.04 (0.02-0.07) |  |
| **Men** | **Multivariable** | **Multivariable** |  |
| Drought | 1.31 (1.04-1.66) | 1.02 (0.46-2.28) |  |
| Age 15-19 | 1 | 1 |  |
| Age 20-24 | 0.95 (0.85-1.06) | 0.86 (0.59-1.24) |  |
| Age 25-29 | 0.74 (0.64-0.87) | 0.51 (0.30-0.86) |  |
| Age 30-34 | 0.64 (0.55-0.76) | 0.64 (0.40-1.03) |  |
| Age 35-39 | 0.57 (0.48-0.67) | 0.55 (0.33-0.92) |  |
| Age 40-44 | 0.56 (0.47-0.66) | 0.87 (0.57-1.33) |  |
| Age 45-49 | 0.50 (0.42-0.59) | 0.76 (0.44-1.33) |  |
| Age 50-54 | 0.51 (0.43-0.60) | 0.93 (0.53-1.64) |  |
| Age 55-59 | 0.53 (0.44-0.65) | 1.05 (0.54-2.06) |  |
| Secondary education or higher | 0.34 (0.31-0.37) | 0.22 (0.16-0.30) |  |
| Received paycheck in last 12 months | 0.71 (0.65-0.76) | 0.66 (0.47-0.92) |  |
| Ever married | 1.73 (1.55-1.92) | 1.17 (0.80-1.71) |  |
| Food support (vs no support) | 1.63 (1.06-2.52) | 0.52 (0.20-1.32) |  |
| Other economic support (vs no support) | 1.77 (1.43-2.18) | 2.64 (1.76-3.95) |  |
| Eswatini | 1 | 1 |  |
| Lesotho | 1.13 (0.91-1.39) | 0.13 (0.07-0.23) |  |
| Tanzania | 1.29 (1.00-1.66) | 0.11 (0.06-0.22) |  |
| Uganda | 1.12 (0.87-1.44) | 0.47 (0.26-0.85) |  |
| Zambia | 1.35 (1.11-1.65) | 0.05 (0.03-0.08) |  |

*Poverty defined as being in the lowest two wealth quintiles

**Supplementary table 8:** Full adjusted weighted odds ratios of sexual behaviours for each wealth quintile

|  |  | **Full adjusted* odds ratios (95% confidence intervals)** | | |  |  |
| --- | --- | --- | --- | --- | --- | --- |
| **Outcome** | **Population** | **Wealth quintile 1** | **Quintile 2** | **Quintile 3** | **Quintile 4** | **Quintile 5** |
| **Transactional sex** | Rural women | 1 | 0.85 (0.75-0.96) | 0.91 (0.79-1.04) | 0.80 (0.68-0.94) | 0.57 (0.41-0.81) |
|  | Urban women | 1 | 1.16 (0.70-1.93) | 0.93 (0.57-1.52) | 0.89 (0.56-1.42) | 0.70 (0.44-1.10) |
|  | Rural men | 1 | 1.02 (0.82-1.28) | 0.99 (0.79-1.23) | 0.75 (0.55-1.04) | 0.66 (0.42-1.04) |
|  | Urban men | 1 | 1.01 (0.50-2.07) | 0.71 (0.33-1.55) | 0.69 (0.38-1.24) | 0.53 (0.30-0.92) |
| **High-risk sex** | Rural women | 1 | 1.22 (1.05-1.41) | 1.16 (0.99-1.36) | 1.18 (0.96-1.46) | 1.14 (0.79-1.65) |
|  | Urban women | 1 | 1.41 (0.79-2.54) | 2.22 (1.30-3.79) | 1.55 (0.94-2.56) | 1.45 (0.89-2.37) |
|  | Rural men | 1 | 1.24 (1.08-1.43) | 1.32 (1.12-1.56) | 1.34 (1.12-1.61) | 1.22 (0.93-1.59) |
|  | Urban men | 1 | 1.73 (1.11-2.71) | 1.59 (1.01-2.48) | 1.63 (1.04-2.54) | 1.06 (0.68-1.66) |
| **Intergenerational sex** | Rural women | 1 | 1.02 (0.92-1.13) | 1.12 (1.00-1.25) | 1.05 (0.91-1.21) | 1.34 (1.08-1.65) |
|  | Urban women | 1 | 0.86 (0.53-1.38) | 1.20 (0.80-1.80) | 1.06 (0.70-1.60) | 1.07 (0.71-1.62) |
|  | Rural men | 1 | 0.91 (0.79-1.06) | 1.03 (0.89-1.19) | 0.91 (0.75-1.10) | 0.94 (0.72-1.23) |
|  | Urban men | 1 | 1.23 (0.76-1.99) | 1.70 (1.09-2.63) | 1.53 (1.03-2.27) | 1.48 (0.99-2.21) |

***** Analyses were adjusted for receipt of food or other economic support, secondary education or higher, having received a paycheck in the previous 12 months, ever having been married, age, and survey country.

**Supplementary table 9:** Full adjusted model results for sexual behaviours and recent HIV

|  | **Women** |  |
| --- | --- | --- |
|  | **Rural** | **Urban** |
|  | **aOR (95% CI)** | **aOR (95% CI)** |
| Transactional sex | 1.73 (0.71-4.18) | 1.61 (0.63-4.10) |
| High-risk sex | 3.40 (1.34-8.59) | 2.86 (1.30-6.32) |
| Intergenerational sex | 1.66 (0.81-3.42) | 2.60 (1.22-5.58) |
| Eswatini & viraemia interaction | 1.16 (1.11-1.21) | 1.06 (0.97-1.16) |
| Lesotho & viraemia interaction | 1.14 (1.09-1.19) | 1.09 (1.04-1.14) |
| Tanzania & viraemia interaction | 1.16 (1.07-1.26) | 0.75 (0.57-0.97) |
| Uganda & viraemia interaction | 1.19 (1.06-1.34) | 1.02 (0.86-1.22) |
| Zambia & viraemia interaction | 1.12 (1.06-1.19) | 1.11 (1.04-1.18) |
| Age (per 10-year increase) | 1.15 (0.90-1.47) | 1.09 (0.86-1.40) |
|  | **Men** |  |
|  | **Rural** | **Urban** |
|  | **aOR (95% CI)** | **aOR (95% CI)** |
| Transactional sex | 0.97 (0.20-4.66) | 0.35 (0.04-3.09) |
| High-risk sex | 1.39 (0.39-4.98) | 3.81 (0.85-17.12) |
| Intergenerational sex | 1.84 (0.68-4.99) | 1.92 (0.47-7.83) |
| Eswatini & viraemia interaction | 1.09 (0.99-1.21) | 1.16 (1.06-1.26) |
| Lesotho & viraemia interaction | 1.14 (1.08-1.20) | 1.08 (1.01-1.16) |
| Tanzania & viraemia interaction | 1.14 (0.97-1.34) | 0.65 (0.29-1.44) |
| Uganda & viraemia interaction | 1.23 (1.08-1.42) | 1.19 (0.96-1.47) |
| Zambia & viraemia interaction | 1.05 (0.92-1.20) | 1.01 (0.88-1.15) |
| Age (per 10-year increase) | 1.52 (1.15-2.00) | 1.38 (0.86-2.20) |

**Supplementary table 10:** Sensitivity analyses for the association between drought and recent HIV

|  | **Fully adjusted* odds ratios (95% confidence intervals)** | | |
| --- | --- | --- | --- |
| **Subgroup** | **Main analysis** | **Stricter definition of droughtⱡ** | **No survey weighting** |
| Rural women | 2.10 (1.17-3.77) | 2.46 (1.38-4.42) | 2.20 (1.29-3.74) |
| Urban women | 0.74 (0.29-1.88) | 0.57 (0.26-1.27) | 0.72 (0.38-1.36) |
| Rural men | 0.52 (0.19-1.45) | 1.07 (0.39-2.91) | 1.13 (0.50-2.58) |
| Urban men | 1.47 (0.40-5.45) | 0.57 (0.26-1.27) | 0.97 (0.55-1.70) |

* Adjusted for age, an interaction between survey country and regional viraemia, secondary education or higher, having received a pay check in the last 12 months, having ever been married, receipt of food or other economic support, severe food insecurity, wealth quintile, transactional sex, high-risk sex, and intergenerational sex.

ⱡ Using the 10^th^ percentile as a cut-off rather than the 15^th^ percentile.

**Supplementary table 11:** Full adjusted weighted odds ratios of having recently acquired HIV*, with age and wealth quintile categorised

|  | **Women** |  | **Men** |  |
| --- | --- | --- | --- | --- |
|  | **Rural** | **Urban** | **Rural** | **Urban** |
| Drought | 2.13 (1.20-3.80) | 0.72 (0.28-1.85) | 0.53 (0.19-1.45) | CNA |
| Age 15-19 | 1 | 1 | 1 | CNA |
| Age 20-24 (vs 15-19) | 0.97 (0.22-4.30) | 1.75 (0.65-4.76) | 12.69 (2.12-76.10) | CNA |
| Age 25-29 (vs 15-19) | 0.96 (0.18-5.01) | 1.11 (0.34-3.60) | 6.20 (0.61-62.99) | CNA |
| Age 30-34 (vs 15-19) | 1.90 (0.32-11.43) | 1.75 (0.53-5.78) | 9.43 (1.01-88.43) | CNA |
| Age 35-39 (vs 15-19) | 0.77 (0.12-4.87) | 1.37 (0.34-5.61) | 11.43 (0.95-138.19) | CNA |
| Age 40-44 (vs 15-19) | 1.31 (0.19-9.03) | 1.03 (0.28-3.84) | 14.45 (1.45-144.09) | CNA |
| Age 45-49 (vs 15-19) | 1.61 (0.25-10.34) | 0.05 (0.01-0.49) | 7.73 (0.52-114.76) | CNA |
| Age 50-54 (vs 15-19) | 2.21 (0.31-15.77) | 0.36 (0.05-2.50) | 26.98 (2.47-294.44) | CNA |
| Age 55-59 (vs 15-19) | 0.39 (0.03-4.75) | 1.40 (0.17-11.88) | 0.00 (0.00-2.36E08<) | CNA |
| Eswatini and regional viraemia interaction | 1.15 (1.09-1.22) | 1.05 (0.95-1.17) | 1.11 (1.00-1.24) | CNA |
| Lesotho and regional viraemia interaction | 1.08 (1.03-1.14) | 1.10 (1.03-1.18) | 1.17 (1.11-1.25) | CNA |
| Tanzania and regional viraemia interaction | 1.18 (1.09-1.29) | 0.70 (0.53-0.93) | 1.14 (0.97-1.34) | CNA |
| Uganda and regional viraemia interaction | 1.19 (1.07-1.33) | 0.98 (0.81-1.18) | 1.21 (1.05-1.40) | CNA |
| Zambia and regional viraemia interaction | 1.09 (1.01-1.17) | 1.13 (1.05-1.21) | 1.08 (0.95-1.22) | CNA |
| Secondary education or higher | 1.64 (0.71-3.77) | 0.71 (0.30-1.65) | 1.44 (0.62-3.37) | CNA |
| Received paycheck in last 12 months | 1.22 (0.57-2.60) | 2.35 (1.35-4.11) | 1.55 (0.72-3.35) | CNA |
| Ever married | 1.79 (0.42-7.68) | 2.07 (0.99-4.34) | 7.32 (1.09-49.14) | CNA |
| No support | 1 | 1 | 1 | CNA |
| Food support (vs no support) | 0.33 (0.12-0.90) | 0.00 (0.00-0.33) | 3.85 (0.50-29.66) | CNA |
| Other economic support (vs no support) | 0.55 (0.23-1.27) | 2.94 (1.07-8.11) | 1.08 (0.23-4.95) | CNA |
| Severe food insecurity | 2.15 (0.91-5.03) | 1.40 (0.56-3.48) | 1.50 (0.53-4.23) | CNA |
| Wealth quintile 1 | 1 | 1 | 1 | CNA |
| Wealth quintile 2 (vs 1) | 0.47 (0.18-1.23) | 4.31 (0.50-36.96) | 1.27 (0.45-3.58) | CNA |
| Wealth quintile 3 (vs 1) | 1.14 (0.55-2.40) | 2.85 (0.34-23.88) | 0.96 (0.37-2.49) | CNA |
| Wealth quintile 4 (vs 1) | 0.57 (0.21-1.53) | 3.32 (0.41-26.71) | 0.65 (0.17-2.46) | CNA |
| Wealth quintile 5 (vs 1) | 0.04 (0.00-0.29) | 2.25 (0.27-19.08) | 1.12 (0.26-4.88) | CNA |
| Transactional sex | 1.58 (0.62-3.98) | 1.32 (0.50-3.46) | 0.79 (0.17-3.80) | CNA |
| High-risk sex | 3.45 (1.36-8.71) | 2.81 (1.29-6.14) | 1.38 (0.39-4.87) | CNA |
| Intergenerational sex | 1.54 (0.72-3.31) | 2.17 (0.99-4.76) | 1.68 (0.66-4.30) | CNA |

CNA: Convergence not achieved.

Regional viraemia is defined as the percentage of individuals in the enumeration area with unsuppressed HIV-1 viral loads (the odds ratio is per percentage increase).

*Among women and men testing negative for HIV antibodies or testing positive with evidence of recent HIV infection

**In depth comparison with other literature**

Few analyses have investigated the associations between drought and HIV incidence or prevalence. Burke et al used Demographic Health Survey (DHS) data to examine the associations between local rainfall shocks and HIV prevalence, finding that infection rates in rural areas where HIV is endemic increase by 11% for every recent drought(8). Using the Lesotho PHIA survey, Low et al also found that drought in rural areas was associated with higher HIV prevalence and riskier sexual behaviour in young women(9). Similarly, Treibich et al’s analysis of Malawi’s DHS data found that experiencing drought increased the likelihood that women employed in agriculture engaged in transactional sex, and that a single drought increased HIV prevalence by around 15% among men and women(10). Austin et al used structural equation modelling to demonstrate that droughts are indirect predictors, via factors such as food insecurity, of the percentage of a country’s population living with HIV who are women(11). Lastly, Epstein et al used multi-country DHS data to show that experiencing droughts was associated with higher odds of condomless sex and lower odds of HIV testing in the prior year(12).

Studies in SSA have looked at the associations between related variables in the theoretical framework linking drought and HIV(13). This includes the relationships between drought and measures of wealth, poverty and sexual behaviours, and the influence of these sexual behaviours on HIV incidence or prevalence. Other literature has found that droughts are associated with extreme poverty in SSA(14), whilst the World Bank has estimated that climate change, including droughts, will lead to millions of people in SSA falling into poverty(15), partially through reliance on subsistence agriculture(16).

Poverty has been shown to impede cognitive function, with people making poorer decisions in times of scarce resources (pre-harvest), compared with when resources are abundant(17). This could also relate to decision-making around sexual risk behaviours. A meta-analysis by Ali and Tadele of studies in SSA found that the poorest men and women had higher odds of not using a condom during their previous risky sexual intercourse(18), which we also observed among men in urban, but not rural, areas. Adair’s study using DHS data on men from 5 countries in SSA found condom use at last intercourse to increase with increasing wealth(19), whilst another multi-country study by Phiri et al found that richer men were more likely to report using condoms, as well as reporting more sexual partners(20). Various studies in South Africa have looked at whether wealth is associated with concurrent sexual partners, an outcome we did not investigate. Kenyon et al’s paper found that poorer men were more likely than their wealthier counterparts to report concurrence(21), whilst Mah et al’s study on younger men(22) and Kenyon and Coleblunders’ study on men and women(23) did not observe this association. A study among young women in Zimbabwe found that lower socio-economic position was associated with increased transactional sex and having older sexual partners(24). We found similar results for transactional sex, but found that higher wealth was associated with increased intergenerational sex, perhaps due to the different setting. A study among women in Kenya found that the odds of transactional sex and unprotected casual sex were higher in the lowest wealth quintile compared to some, but not all, other quintiles(25). This is potentially due to losing statistical power by treating wealth as a categorical rather than continuous variable(25). Meanwhile, various studies have found that food insecurity is associated with increased risky sexual behaviours in SSA(1, 24, 26-28). A systematic review and meta-analysis found that transactional sex was associated with prevalent HIV infections(29). Other research has found that intergenerational sex is predictive of prevalent HIV infection among young women(30), and that, for young women, older partner age is associated with increased odds of recent HIV infection(31).

**Supplementary table 12:** STrengthening the Reporting of OBservational studies in Epidemiology (STROBE) checklist.

|  | Item No | Recommendation | Page |
| --- | --- | --- | --- |
| **Title and abstract** | 1 | (*a*) Indicate the study’s design with a commonly used term in the title or the abstract | 1 (title) |
|  |  | (*b*) Provide in the abstract an informative and balanced summary of what was done and what was found | 2 (abstract) |
| Introduction | | |  |
| Background/rationale | 2 | Explain the scientific background and rationale for the investigation being reported | 4 (paragraphs 1-3) |
| Objectives | 3 | State specific objectives, including any prespecified hypotheses | 4 (paragraph 4) |
| Methods | | |  |
| Study design | 4 | Present key elements of study design early in the paper | 5 (Data and procedures) |
| Setting | 5 | Describe the setting, locations, and relevant dates, including periods of recruitment, exposure, follow-up, and data collection | 5 (Data and procedures) |
| Participants | 6 | (*a*) Give the eligibility criteria, and the sources and methods of selection of participants | 5 (Data and procedures) and appendix pages 4-6 |
| Variables | 7 | Clearly define all outcomes, exposures, predictors, potential confounders, and effect modifiers. Give diagnostic criteria, if applicable | 5-6 (Variables and Statistical analyses) |
| Data sources/ measurement | 8 | For each variable of interest, give sources of data and details of methods of assessment (measurement). Describe comparability of assessment methods if there is more than one group | 5-6 (Variables and Statistical analyses) |
| Bias | 9 | Describe any efforts to address potential sources of bias | 6 (Statistical analyses; re weighting) |
| Study size | 10 | Explain how the study size was arrived at | Supplementary table 1 (PHIA survey sampling frames and sample sizes) |
| Quantitative variables | 11 | Explain how quantitative variables were handled in the analyses. If applicable, describe which groupings were chosen and why | 5-6 (Variables and Statistical analyses) |
| Statistical methods | 12 | (*a*) Describe all statistical methods, including those used to control for confounding | 6 (Statistical analyses) |
|  |  | (*b*) Describe any methods used to examine subgroups and interactions | 5 (Variables) |
|  |  | (*c*) Explain how missing data were addressed | 7 (Statistical analyses) |
|  |  | (*d*) If applicable, describe analytical methods taking account of sampling strategy | 6 (Statistical analyses; re weighting) |
|  |  | (*e*) Describe any sensitivity analyses | 5 (Variables) |
| Results | | |  |
| Participants | 13 | (a) Report numbers of individuals at each stage of study—eg numbers potentially eligible, examined for eligibility, confirmed eligible, included in the study, completing follow-up, and analysed | 8 (Results) and supplementary table 5 (PHIA survey response rates) |
|  |  | (b) Give reasons for non-participation at each stage | 8 (Results) and supplementary table 5 (PHIA survey response rates) |
|  |  | (c) Consider use of a flow diagram | Flow diagram information for individual surveys are linked to on page 3 of the appendix. |
| Descriptive data | 14 | (a) Give characteristics of study participants (eg demographic, clinical, social) and information on exposures and potential confounders | Page 8 (Results) and Table 1 (Weighted demographic characteristics by wealth quintile, stratified by gender.) |
|  |  | (b) Indicate number of participants with missing data for each variable of interest | 9 (Sexual behaviours and recent HIV) |
| Outcome data | 15 | Report numbers of outcome events or summary measures | Table 1 (Weighted demographic characteristics by wealth quintile, stratified by gender.) and table 4 (Associations between having recently acquired HIV and sexual behaviours among HIV-negative women and men, separately for rural and urban areas (weighted).) |
| Main results | 16 | (*a*) Give unadjusted estimates and, if applicable, confounder-adjusted estimates and their precision (eg, 95% confidence interval). Make clear which confounders were adjusted for and why they were included | Tables 2-5. |
|  |  | (*b*) Report category boundaries when continuous variables were categorized | NA |
|  |  | (*c*) If relevant, consider translating estimates of relative risk into absolute risk for a meaningful time period | NA |
| Other analyses | 17 | Report other analyses done—eg analyses of subgroups and interactions, and sensitivity analyses | 9 (Sexual behaviours and recent HIV & Drought and recent HIV) |
| Discussion | | |  |
| Key results | 18 | Summarise key results with reference to study objectives | 10 (first paragraph) |
| Limitations | 19 | Discuss limitations of the study, taking into account sources of potential bias or imprecision. Discuss both direction and magnitude of any potential bias | 11 (Strengths and limitations) |
| Interpretation | 20 | Give a cautious overall interpretation of results considering objectives, limitations, multiplicity of analyses, results from similar studies, and other relevant evidence | 12 (Implications) |
| Generalisability | 21 | Discuss the generalisability (external validity) of the study results | 11 (Strengths and limitations) |
| Other information | | |  |
| Funding | 22 | Give the source of funding and the role of the funders for the present study and, if applicable, for the original study on which the present article is based | 2 (Abstract) |

**References**

1. Low A, Gummerson E, Schwitters A, Bonifacio R, Teferi M, Mutenda N, et al. Food insecurity and the risk of HIV acquisition: findings from population-based surveys in six sub-Saharan African countries (2016-2017). Bmj Open. 2022;12(7).

2. Pendergrass AG, Knutti R, Lehner F, Deser C, Sanderson BM. Precipitation variability increases in a warmer climate. Sci Rep. 2017;7(1):17966.

3. ICAP at Columbia. Population-based HIV Impact Assessment (PHIA) Data Use Manual. New York, NY; 2019 July 2019.

4. Filmer D, Pritchett LH. Estimating wealth effects without expenditure data - Or tears: An application to educational enrollments in states of India. Demography. 2001;38(1):115-32.

5. Rutstein S, Kiersten J. The DHS Wealth Index. Calverton, Maryland: ORC Macro;; 2004.

6. Howe LD, Hargreaves JR, Huttly SR. Issues in the construction of wealth indices for the measurement of socio-economic position in low-income countries. Emerg Themes Epidemiol. 2008;5:3.

7. Kaiser BN, Hruschka D, Hadley C. Measuring material wealth in low-income settings: A conceptual and how-to guide. Am J Hum Biol. 2017;29(4).

8. Burke M, Gong E, Jones K. Income Shocks and HIV in Africa. Econ J. 2015;125(585):1157-89.

9. Low AJ, Frederix K, McCracken S, Manyau S, Gummerson E, Radin E, et al. Association between severe drought and HIV prevention and care behaviors in Lesotho: A population-based survey 2016-2017. Plos Med. 2019;16(1).

10. Treibich C, Bell E, Blanc E, Lepine A. From a drought to HIV: An analysis of the effect of droughts on transactional sex and sexually transmitted infections in Malawi. Ssm-Popul Hlth. 2022;19.

11. Austin KF, Noble MD, Berndt VK. Drying Climates and Gendered Suffering: Links Between Drought, Food Insecurity, and Women's HIV in Less-Developed Countries. Soc Indic Res. 2021;154(1):313-34.

12. Epstein A, Nagata JM, Ganson KT, Nash D, Saberi P, Tsai AC, et al. Drought, HIV Testing, and HIV Transmission Risk Behaviors: A Population-Based Study in 10 High HIV Prevalence Countries in Sub-Saharan Africa. Aids Behav. 2022.

13. UNAIDS. Climate Change and AIDS: A Joint Working Paper. 2008.

14. Azzarri C, Signorelli S. Climate and poverty in Africa South of the Sahara. World Dev. 2020;125.

15. Hallegate S, Bangalore M, Bonzanigo L, Fay M, Kane T, Narloch U, et al. Shock Waves: Managing the Impacts of Climate Change on Poverty. Washington, DC.: World Bank,; 2016.

16. Hope KR. Climate change and poverty in Africa. Int J Sust Dev World. 2009;16(6):451-61.

17. Mani A, Mullainathan S, Shafir E, Zhao JY. Poverty Impedes Cognitive Function. Science. 2013;341(6149):976-80.

18. Ali R, Tadele A. Risky Sexual Behavior across Extremes of Wealth in sub-Saharan Africa: A Meta-Analysis of Demographic and Health Surveys. Ethiop J Health Sci. 2021;31(1):159-66.

19. Adair T. Men's condom use in higher-risk sex: Trends in five sub-Saharan African countries. Journal of Population Research. 2208;25:51-62.

20. Phiri M, Lemba M, Chomba C, Kanyamuna V. Examining differentials in HIV transmission risk behaviour and its associated factors among men in Southern African countries. Hum Soc Sci Commun. 2022;9(1).

21. Kenyon CR, Osbak K, Buyze J, Johnson S, van Lankveld J. Variations of Sexual Scripts Relating to Concurrency by Race, Class, and Gender in South Africa. J Sex Res. 2015;52(8):878-86.

22. Mah TL. Prevalence and Correlates of Concurrent Sexual Partnerships Among Young People in South Africa. Sex Transm Dis. 2010;37(2):105-8.

23. Kenyon C, Colebunders R. Correlates of concurrency among young people in Carletonville, South Africa. Sahara J-J Soc Asp H. 2015;12(1):51-8.

24. Pascoe SJS, Langhaug LF, Mavhu W, Hargreaves J, Jaffar S, Hayes R, et al. Poverty, Food Insufficiency and HIV Infection and Sexual Behaviour among Young Rural Zimbabwean Women. Plos One. 2015;10(1).

25. Muchomba FM, Wang JSH, Agosta LM. Women's land ownership and risk of HIV infection in Kenya. Soc Sci Med. 2014;114:97-102.

26. Miller CL, Bangsberg DR, Tuller DM, Senkungu J, Kawuma A, Frongillo EA, et al. Food Insecurity and Sexual Risk in an HIV Endemic Community in Uganda. Aids Behav. 2011;15(7):1512-9.

27. McCoy SI, Ralph LJ, Njau PF, Msolla MM, Padian NS. Food Insecurity, Socioeconomic Status, and HIV-Related Risk Behavior Among Women in Farming Households in Tanzania. Aids Behav. 2014;18(7):1224-36.

28. Weiser SD, Leiter K, Bangsberg DR, Butler LM, Korte FPD, Hlanze Z, et al. Food insufficiency is associated with high-risk sexual behavior among women in Botswana and Swaziland. Plos Med. 2007;4(10):1589-98.

29. Wamoyi J, Stobeanau K, Bobrova N, Abramsky T, Watts C. Transactional sex and risk for HIV infection in sub-Saharan Africa: a systematic review and meta-analysis. J Int Aids Soc. 2016;19.

30. Bajunirwe F, Semakula D, Izudi J. Risk of HIV infection among adolescent girls and young women in age-disparate relationships in sub-Saharan Africa. Aids. 2020;34(10):1539-48.

31. Ayton S, Schwitters A, Mantell JE, Nuwagaba-Biribonwoha H, Hakim A, Hoffman S, et al. Male partner age, viral load, and HIV infection in adolescent girls and young women: Evidence from eight sub-Saharan African countries. Aids. 2022.
